# Statistical Mechanics and Thermodynamics of Viral Evolution

**DOI:** 10.1371/journal.pone.0137482

**Published:** 2015-09-30

**Authors:** Barbara A. Jones, Justin Lessler, Simone Bianco, James H. Kaufman

**Affiliations:** 1 Almaden Research Center, IBM, San Jose, California, United States of America; 2 Department of Epidemiology, Johns Hopkins Bloomberg School of Public Health, Baltimore, Maryland, United States of America; The University of Melbourne, AUSTRALIA

## Abstract

This paper uses methods drawn from physics to study the life cycle of viruses. The paper analyzes a model of viral infection and evolution using the "grand canonical ensemble" and formalisms from statistical mechanics and thermodynamics. Using this approach we enumerate all possible genetic states of a model virus and host as a function of two independent pressures–immune response and system temperature. We prove the system has a real thermodynamic temperature, and discover a new phase transition between a positive temperature regime of normal replication and a negative temperature “disordered” phase of the virus. We distinguish this from previous observations of a phase transition that arises as a function of mutation rate. From an evolutionary biology point of view, at steady state the viruses naturally evolve to distinct quasispecies. This paper also reveals a universal relationship that relates the order parameter (as a measure of mutational robustness) to evolvability in agreement with recent experimental and theoretical work. Given that real viruses have finite length RNA segments that encode proteins which determine virus fitness, the approach used here could be refined to apply to real biological systems, perhaps providing insight into immune escape, the emergence of novel pathogens and other results of viral evolution.

## Introduction

Viruses are microscopic subcellular objects that infect cells of living organisms across all six kingdoms of life [1]. Because viruses require host cellular machinery to replicate [2], a common set of steps must occur for the reproduction of most viruses. First, the virus must enter the cell, which can occur through membrane fusion, endocytosis, or genetic injection [3]. During the replication process, tens to thousands of progeny are produced [2]. While the fidelity of the replication process varies between viruses, for most, particularly RNA viruses, the mutation rate is quite high [2]. Finally, progeny exit the cell (via budding, apoptosis, or exocytosis), in many cases killing the cell in the process [2]. The generally high levels of genetic variability created during replication lead to rapid “exploration” of genetic sequence space, allowing the virus to evade the host immune system, overcome environmental challenges such as antiviral drugs, and perhaps even adapt to new host species [4–6]. While even single cell organisms have an innate immune response, viral evolution becomes particularly important when viruses attempt to evade the adaptive immune system of humans and other vertebrates [2,7]. Successful viruses all must survive host defense mechanisms, compete to infect host cells, reproduce, and eventually pass to other hosts [2,8], though an immense variety of strategies are used to accomplish these tasks.

Recent technical advances in genome sequencing have revealed the enormous genetic diversity of RNA virus populations during infection [9], which is triggered by large population size and low replication fidelity. Information about mutation distributions during evolution has proven to be helpful in assessing the intricate mechanisms of viral reproduction [10,11]. Moreover, new insights into the tradeoff between mutational robustness, loosely defined as the *invariance of the phenotype to genetic insults*, and evolvability, *the capability of the viral species to adapt to new environments*, are emerging, supported by a wealth of new data [12,13].

However, it may not always be necessary, or even advisable, to capture the full intricacies of this system in useful models of viral evolution and dynamics. Highly simplified models may still reveal important principles about the behavior of viral populations. For example, Alonso and Fort measured thermodynamic observables to analyze a phase transition observed in a model of RNA virus error catastrophe [14–16]. In analogy to Bose condensation they derive an order parameter to characterize two phases separated by the error catastrophe phase transition. The error catastrophe literature demonstrates the importance of mutation rate and reveals a phase transition due to information loss at large rate [14–22]. Nowak and May consider the transition from sustained infection to elimination of infection as a function of basic reproductive ratios for various mutant strains [16]. In the current work we demonstrate a way to compute the steady state solutions for evolving viral quasispecies on a fitness landscape determined by *two independent and competing energy terms*, temperature and immunity.

Statistical mechanics allows physicists to describe the macroscopic characteristics of a multi-particle system based on microscopic properties [23–25]. Given a large collection of molecules or atomic particles, it is possible to use probability theory to define macroscopic properties in terms of thermodynamic quantities such as system heat, energy, and entropy [23–25]. These macroscopic properties are determined by an “ensemble” of all “microstates” of the collection, along with the probabilities associated with each microstate. If a simple model of viral replication, transmission and evolution can be developed that lends itself to such analysis, it may serve as a foundation on which to develop a powerful theory to describe the general behavior of viral systems using the same key concepts used in statistical mechanics.

## Methods

In this paper we present a model of viral replication and evolution within a single host and the analytic theory required to find steady-state solutions for this system. We then describe the steps used to solve the analytic equations and the methods used. Finally, we study the thermodynamics and statistical mechanics of the viral evolution model. We calculate thermodynamic quantities such as entropy, an order parameter, specific heat, energy, and properties of viral population dynamics such as host cell occupancy and viral load in the environment.

### Viral Infection as Energy Barriers

Viruses replicate and transmit by a complex multi-step process. For an influenza virion to infect a cell and replicate, it must bind to receptors on the cellular membrane; induce endocytosis; release ribonucleoprotein (vRNP) complexes into the cytoplasm; vRNPs must be imported into the nucleus where replication can occur; and viral offspring must leave the cell through viral budding [26]. At each step there is some probability of failure, and the more fit the virus, the lower this probability [27,28]. We abstract this process as crossing two symmetric barriers, one for infecting the cell, and one for replication and exit.

**Fig 1:**
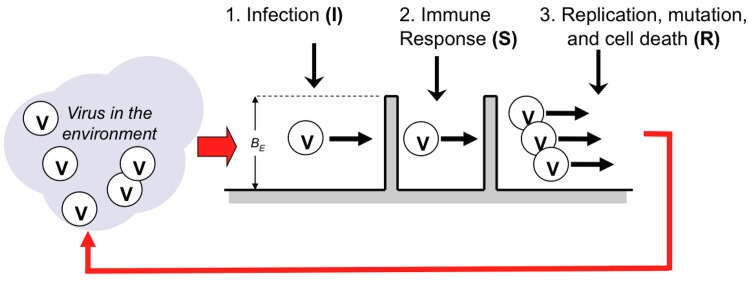
Model of an Idealized Virus Life Cycle. The barrier height (B_E_) is equal to the number of mismatches of virus to a target. Viruses with different numbers of genetic matches will see barriers of different height. The probability of a virus passing is based on an activated Arrhenius model.

The virus has some fitness for crossing these barriers (Fig 1), characterized by a probability of successes which depends on viral fitness and system “temperature”, and is computed using an activated Arrhenius form [23]:

$$e_{i}=\exp\left(f_{i}\;/\;T\right)$$ (1)

where *e*_*i*_ is the probability of successfully crossing the barrier, *f*_*i*_ is viral fitness, and *T* is the system temperature. At this point one can view T as a parameter that governs how discriminating the barrier is between viruses with different numbers of “matches” to some target receptor. In classical chemistry the barrier height is a function of both reactants and products, while the temperature is a property of the reactants only (viruses in this case). When there is a distribution of energies for the reactants, *k*_*B*_T is the average energy of the most probable distribution. In this model temperature changes the discrimination of the barriers to infection and reproduction. At low temperature viruses with a high match to the target are favored. At very high temperature, virtually all viruses of any match are able to infect and reproduce. As we will see, raising the temperature increases the fraction of viable virions within a quasispecies distribution. We will later demonstrate that temperature in this model is not only a tuning parameter, but also the thermodynamic temperature for the system, providing a distribution of energies for the viruses, which naturally form quasispecies distributions. We will also derive the effective Boltzmann constant relating temperature and the observed energy scales [23–26].

In viral entry, a protein creates a receptor on the viral envelope that binds to complementary receptors on a target cell membrane. We do not intend to model the complex interactions between these receptors. Instead, we abstract the fitness of a virus by how well the genetic letters (an abstraction of amino acids) in a virus’s genome match an idealized *target* genetic sequence. The receptor in a real virus is a sub-region of the binding protein (providing many degrees of freedom). To capture these degrees of freedom we require that a sub-region of a virus “gene” matches a complementary genetic (receptor) target in the cell. Specifically, we define a target sequence of 50 letters in length associated with the host cell, and each virus is assigned a genome of 100 letters (i.e., 300 bases). As with real amino acids, letters are the phenotypic representation of a codon of three underlying bases (*A*,*C*,*G*, and *U/T*) in a redundant genetic code (see S1 Appendix for details). We define *m* as the number of *matches* between host and target sequences at the alignment that minimizes the total number of mismatches (but still completely overlays the target). This model is not intended to capture the complex interactions between proteins based on their real shapes. Instead, viral fitness in this abstraction is completely characterized by the difference between the number of matches and the length of the genome (i.e., *f*_*i*_ = *f*_*m*_ = −(50 − *m*)). Once the number of matches to the target is determined for a particular virion, it is fixed for the life of that virion.

Then, given Eq (1), the probability of a successful barrier crossing is:

$$e_{m}=\exp\left(-\left(50-m\right)\;/\;T\right)$$ (2)

On each replication there is some probability of mutation in a given base, allowing the distribution of viruses to change or evolve over time.

### The Viral Life Cycle

**Fig 2:**
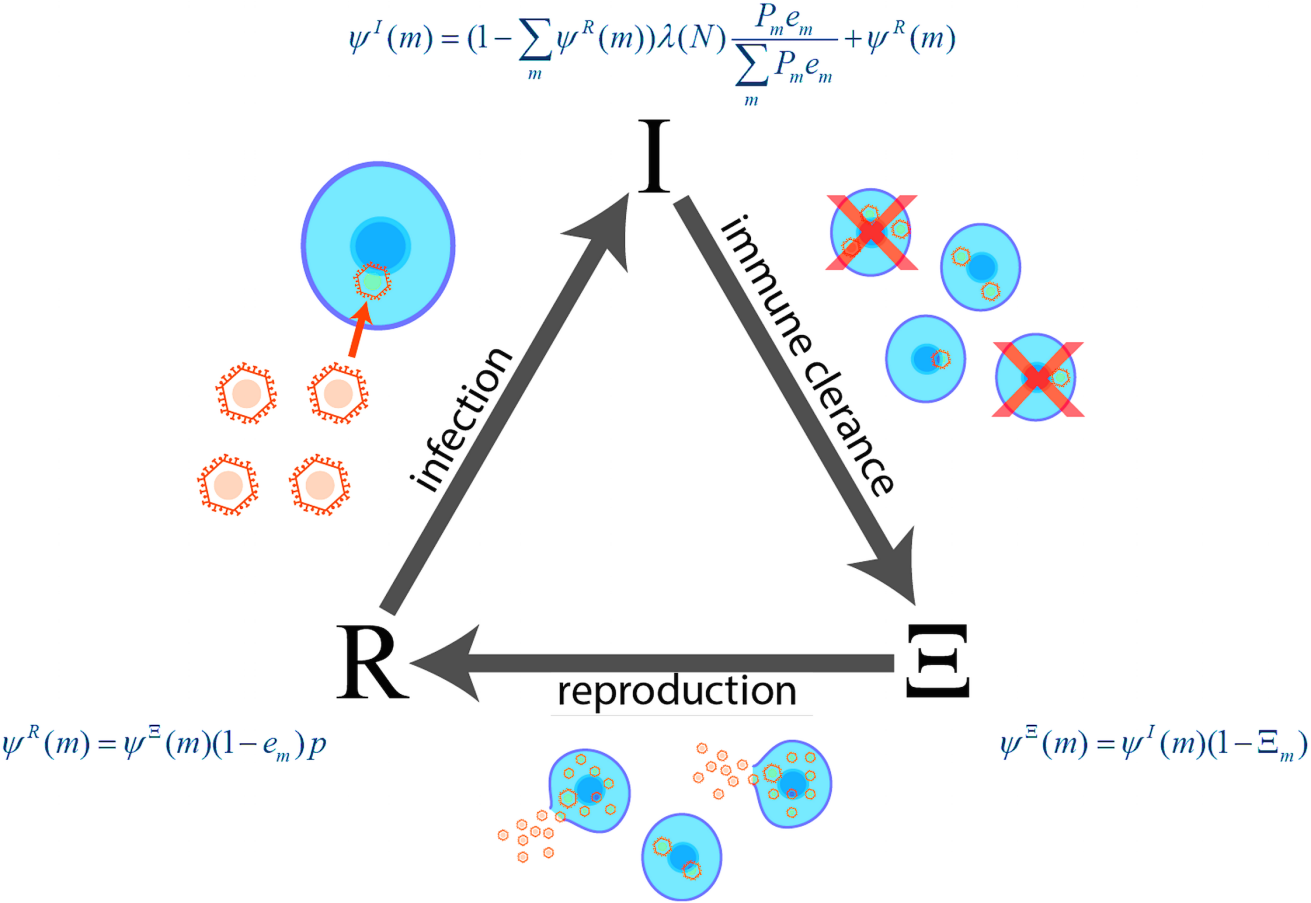
Virus Life Cycle. The changing states of all viruses must be computed self-consistently over the entire virus life cycle. The figure shows three important stages of the model virus life cycle: (I) Infection (entering the cell), (Ξ) Immune Clearance, and (R) Reproduction and exiting the cell. Also shown are the equations for cell occupancy at each stage.

In our simplified model of viral infection and replication the system of viruses passes through three stages in discrete generations (Fig 2). Free viruses first infect cells, passing into the post-infection stage, *I*. Some proportion of infected cells are then “killed” by the immune system, and instantly replaced by uninfected cells, and we enter the post-immunity stage, *Ξ*. Finally, viruses replicate and exit the cell, and we enter the post-reproduction/pre-infection stage, *R*. The system state in each stage can be described completely by two interacting sets of variables: the occupation of the host cells, and the distribution of “free” viruses in the environment. The *self-consistent* (steady state) solution for the virus life cycle is one in which each state remains unchanged after completing a full cycle.

We denote the probability that a cell in our model has a virus with given number of matches for each stage as *Ψ* ^*I*^ *(m)*, *Ψ* ^Ξ^ *(m)*, and *Ψ* ^*R*^ *(m)*. Before reproduction, each cell is considered to contain at most a single virion. The number of free viruses is denoted by *N* and the proportion with *m* matches to the target is denoted as *P*_*m*_. Both *N* and *P*_*m*_ are only defined in the post-reproduction stage, after the free virus population is replenished from those intra-cellular viruses that survive reproduction. The equations for cell occupancy at each stage are:

$$\begin{array}{l} \psi^{I}(m)=(1-\sum_{m}\psi^{R}(m))\lambda (N)\frac{P_{m}e_{m}}{\sum_{m}P_{m}e_{m}}+\psi^{R}(m)\\ \psi^{\Xi }(m)=\psi^{I}(m)(1-\Xi_{m})\\ \psi^{R}(m)=\psi^{\Xi }(m)(1-e_{m})p \end{array}$$ (3)

where *λ*(*N*) is the overall infection rate, *Ξ*_*m*_ is the probability that the host immune response kills any virus in the cell with *m* matches, and *p* is the probability that a cell infected by a virus that does not reproduce survives until the next round of replication (cells that reproduce are considered to die, and all dying cells are considered to be instantly replaced with uninfected cells). Eq (3) represent the following: First, if the cell is empty, with probability$(1-\sum_{m}\psi^{R}(m))$, the cell becomes infected. Next, the cell remains occupied if the virus survives the immune response with probability (1- Ξ_m_). Finally, the cell only remains occupied if the virus does not reproduce with probability *(1-e*_*m*_ *)p*. The virus that remains, *Ψ* ^*R*^ *(m)*, continues to occupy the cells when the next round of infection occurs.

In our model there are a finite number of identical target host cells available to infect at any one time, with the infection process proceeding as follows:

1. At any time at most one virus can infect each cell.
2. Each free virus successively attempts to infect the unoccupied cells with a success probability of each attempt of *e*_*m*_.
3. Competition continues until either all cells are occupied or all free viruses have made an attempt.

With these criteria we can analytically derive the overall infection rate, *λ*(*N*), as a function of the number of target host cells and the number of free viruses in the environment *N* (see below).

#### The immune responses

Vertebrate hosts defend themselves from viral infections using both innate and adaptive immune responses [21]. In our model the innate immune response can be considered to be captured by the barrier that viruses must cross to infect and replicate in cells, while we explicitly model the adaptive immune response. In an adaptive immune response, the immune system develops an increasingly strong and specific response to infecting viruses by producing cells and antibodies which recognize and respond to specific viral epitopes (i.e., short sequences of amino acids that identify the virus) [21]. Here we assume that all parts of the virus are exposed to the immune system. Furthermore, we are interested in a steady state solution where the immune system has learned to recognize the target epitopes (not the entire viral genome). In steady state a virus genome matches some part of the target genome. In analogy to adaptive response to a specific set of epitopes, we use this matching sub-region to determine efficiency of immune response in steady state. In particular we represent the ability of the adaptive immune system to kill infected cells as a function of the match between a virion and the target as:

$$\Xi_{m}=A\;/\;(1+e^{-(m-v)/2})$$ (4)

where 0 ≤ *A* ≤ 1 is the maximum immune response, and *v* is the number of matching codons at which the virus achieves 50% efficiency when *A* = 1. In real viruses, the length of epitopes targeted by immune effector cells can vary widely, from as few as 3–4 of critical amino acids for B-cell conformational epitopes, to 8–11 amino acids for T cell epitopes (for example) [29]. Here we chose an intermediate value and ν = 6 as a typical epitope length. The results and conclusions are not highly sensitive to this choice.

This abstraction is meant to model the steady state response of an adaptive antibody-mediated immunity. In this paper we explore the full range of *A* and *m*. Eq (4), together with Eq (2) and Eq (3), defines the two dimensional fitness landscape in this model.

#### Viral reproduction and mutation

Viral offspring differ from their parent through mutation of individual bases during the replication process. The resulting evolution is an important component of the survival strategy for many viruses, allowing them to evade the immune system and respond to changes in their environment (e.g., the introduction of chemotherapeutic agents). When a virus reproduces, the *actual* reproductive rate (number of offspring per parent) defines the “fecundity”. In our model replication occurs with a fecundity, φ, and offspring have one, and only one, codon mutation per offspring. Mutation to the same amino acid is allowed.

As in the Moran model, single mutation can either reduce the maximal match length by one (*Δm* = −1), increase the maximal match length by one (*Δm* = +1) or leave the maximal match length unchanged (*Δm* = 0) [18,19]. Consider a virus with a maximum of *m*_*0*_ codons matching the organism target genome. We find the sub-region(s) on the virus genome that contain the maximum number of matches, *m*_*0*_, to the target by counting matches for each possible alignment. For a mutation to decrease *m*, two conditions must be true. First, the mutation must occur at a currently matched position in the matching region and must change the expressed amino acid (i.e., must not be a same-sense or silent mutation). Second, there must not be two or more alignments with the same maximal match. For a mutation to increase *m*, it must occur at a non-matching codon in a maximally matching alignment and result in a change to the amino acid at that position to one that matches the target.

The mutation operators take the distribution of viruses that survive the immune response process, and transform it into a distribution of free viruses that exist after reproduction and mutation (Fig 2). In order to calculate the probability distribution, P(m), for the viral state after mutation, we need to calculate the general “transition probability" matrix for changes in the number of matches as a function of m. Assume a virus with a given number of initial matches, m. To calculate the probability that a mutation on this virus will cause a +1, -1, or 0, change in the number of matches we first studied some limiting cases. The transition probability and its slope can be derived analytically at two limits: no (or few) matches and perfect or near perfect matches. Depending on the degeneracy of the target, the values for these limiting cases can vary considerably and have the most variability for viruses with low numbers of matches before mutation. In the case of a highly degenerate target, that is, one with a very limited number of distinct or unique codons, the probability of a single mutation keeping the same number of matches approaches zero in the low initial match limit. Conversely, the probability of a mutation increasing the number of matches goes rapidly (to one) in this limit.

In the opposite case of a target with no repeating codons, the results depend on whether the alphabet is much larger even than the size of the target, or whether the target uses almost all the possible codons. If there are a large number of distinct codons possible in the viruses, beyond the number already in the target, the probability of a mutation keeping the same number of matches approaches 1 in the low-match limit, while the probability of a mutation increasing the number of matches is low for all initial numbers of matches. If almost all the available codons appear in the target, and the viruses contain essentially the same selection of codons that the target does, then the probabilities for keeping the same number of matches and increasing the number of matches by 1 become nearly equal at 1/2 each in the low match limit.

The probability that a mutation *decreases* the number of matches starts at zero for zero initial matches, and typically rises to a value near 0.5 for a complete match with the target, independent of target degeneracy. The probability of a mutation causing no change in the perfectly matching virus is also near 0.5 regardless of target degeneracy.

**Fig 3:**
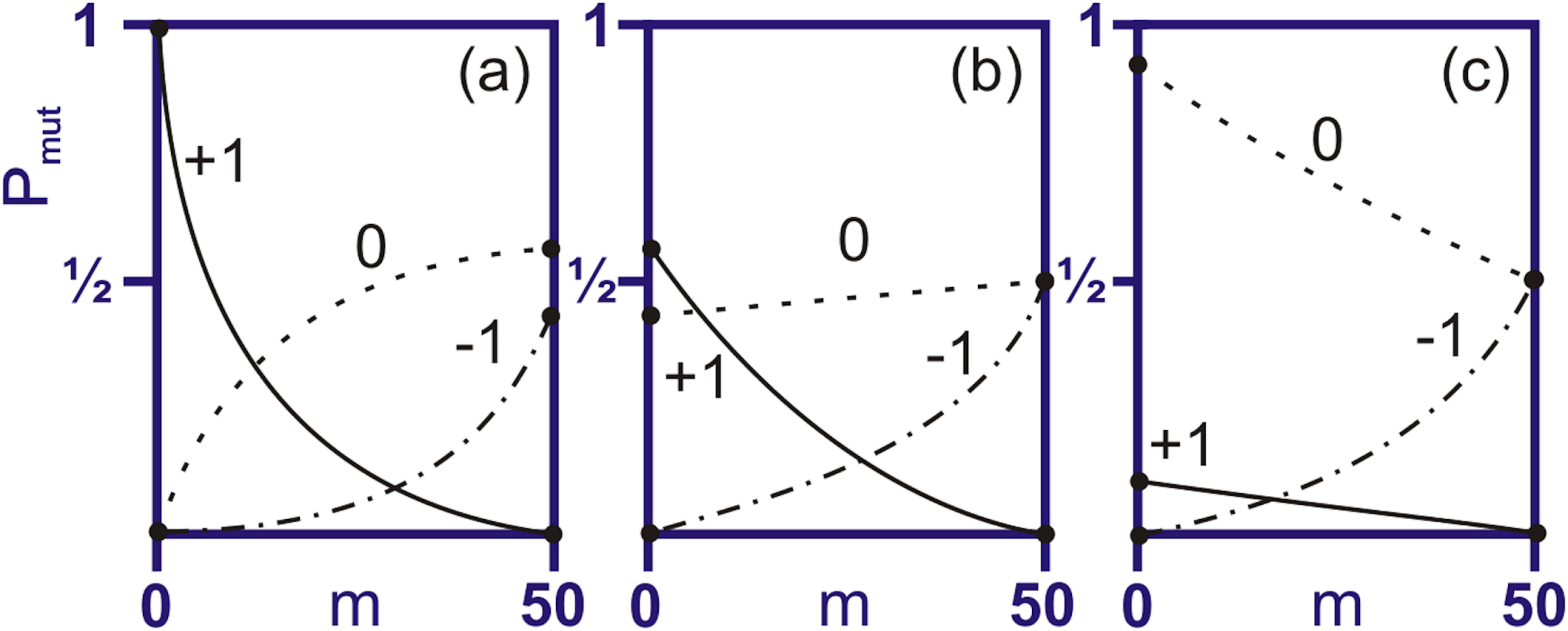
Three Limiting Cases of the Effect of Viral Mutation in a Single Codon. The transition probabilitiy (P_mut_) as a function of the number of matches, m (pre-mutation). Curves labeled ‘+1’ represent the probability of a mutation increasing the number of matches between the virus and target genomes, ‘-1’ the probability of decreasing the number of matches, and ‘0’ the probability of no change. Given alphabet length ‘*a*,’ the figure shows the limiting behavior for: (a) Small *a*, highly degenerate target; (b) *a ≈* target+1, i.e., medium degeneracy; (c) Large *a*, i.e., low target degeneracy.

These general cases are shown in Fig 3. It should be noted that these results hold for matches determined by complete overlay of the target binding sites by the virus. We do not consider alignments where the target extends past the end of the virus giving only partial overlay of the target binding sites. This alternative method would yield different limiting cases, as well as different expressions below for the mutation. For clarity, in our model there exist only v-t+1 allowed alignments, where v and t are the length of virus and target, respectively. (This is in contrast to the ‘extended alignment’ method, not implemented here, which has v+t-1 alignments.) The limiting cases shown in Fig 3 are derived for the specific choice of a virus genome segment exactly twice as long as the target segment (our model system).

For specificity, the target genome segment was defined as:

THISISTHEENTRYTARGETFORREGULARCELLSWAYOFENTERINGIT

This example target uses 16 of the 26 possible codons. It has a maximum degeneracy of 7, and a length of 50. For a virus length of 100, and given this particular target genome segment, we were able to calculate analytically the behavior near the two end points and numerical continuation in between. The mutation probabilities are:

$$P_{mut}(\Delta m=-1)=\omega \frac{m}{100}\left(\frac{1}{1+e^{-(m-10)/2}}\right)$$

$$P_{mut}(\Delta m=+1)=\frac{\omega }{235.45}(e^{4.709(1-m/50)}-1)$$ (5)

$$P_{mut}(\Delta m=0)=1-P_{mut}(\Delta m=+1)-P_{mut}(\Delta m=-1)$$

where *P*_*mut*_ is the probability that a mutation of a virus with m matches will result in a change in m of Δ*m* = 0,±1. The variable *ω*, which we take to be 0.7867, represents the redundancy in the underlying genetic code due to the multiplicity of three-codon combinations that define an amino acid.

**Fig 4:**
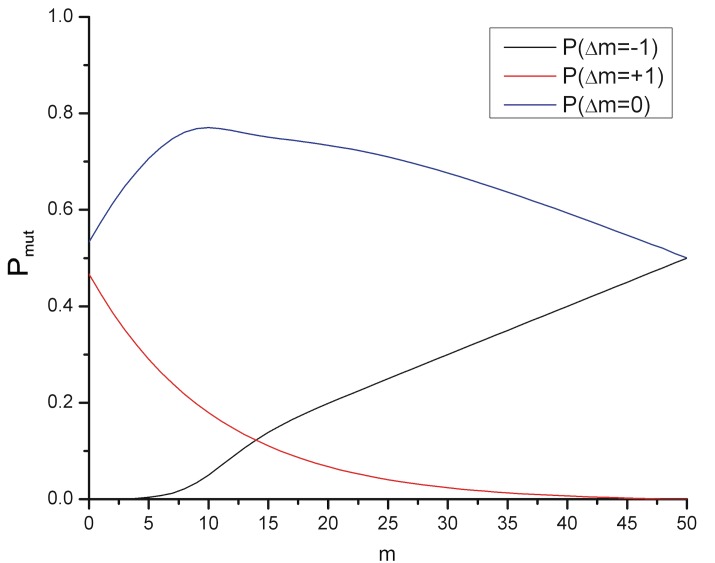
Viral Mutation Probabilities. For our model target, the transition probability (P_mut_) as a function of the number of matches, m (pre-mutation). The figure shows the probability, P_mut_, of a viral mutation causing an increase, decrease, or stasis in the number of matching codons between the virus and the actual target genome (Δm = +1,0,-1).

Note that the sum of the three terms is always one, corresponding to conservation of virus in the mutation process. We plot these mutation probabilities in Fig 4.

We note that our particular target is at the low degeneracy limit, and is part-way between the cases using all the available alphabet vs. those using only a small fraction of it. It thus can be viewed as fairly “challenging” to the virus. We find that changing the length of the virus genome, changing the length of the target, or changing the degeneracies in the codon “alphabet” (without going into trivial regimes or limiting cases of no degeneracy) only rescales temperature without changing the overall behavior or conclusions. In future work it is straightforward to explore other cases in detail.

Note also that many viruses not only mutate, but also recombine. Such viruses may infect individual cells multiple times. This model does not capture these processes. In addition, neither matches nor mismatches in this model should be interpreted as “replication error.” In contrast to the quasispecies model(s) of viral evolution used to study viral error catastrophe [14–22], evolution in this model is a function of two independent and *competing* fitness pressures.

### Self-Consistent Solutions

We solve self-consistently the probability functions of the virus in the host cells {*Ψ* ^*I*^, *Ψ* ^Ξ^, *Ψ* ^*R*^}, the virus distribution in the environment, and the total number of viruses. The 51x51 matrix of inter-related *Ψ(m)*, Eq (3), can be diagonalized analytically to obtain the following:

$$\psi^{I}(m)=\frac{\lambda (N)[P_{m}e_{m}/E]}{[1+\sum_{m'}K_{m'}][1-(1-\Xi_{m})(1-e_{m})p]}$$

$$\psi^{\Xi }(m)=\frac{\lambda (N)[P_{m}e_{m}/E](1-\Xi_{m})}{[1+\sum_{m'}K_{m'}][1-(1-\Xi_{m})(1-e_{m})p]}$$ (6)

$$\psi^{R}(m)=\frac{\lambda (N)[P_{m}e_{m}/E](1-\Xi_{m})(1-e_{m})p}{[1+\sum_{m'}K_{m'}][1-(1-\Xi_{m})(1-e_{m})p]}$$

with

$$K_{m}=\frac{\lambda (N)P_{m}e_{m}(1-\Xi_{m})(1-e_{m})p}{E[1-(1-\Xi_{m})(1-e_{m})p]}$$

where $E=\sum P_{m}e_{m}$ and all other terms are defined as in Eqs (2–4). Eq (6) give the probability that a cell is occupied by virus with *m* matches at each stage of the life cycle (I, Ξ, and *R*). The stable solutions to Eq (6) hold for any given *P*_*m*_ and λ*(N)*, which must also be solved self-consistently.

The limiting case of p = 0 has no virus remaining in the cells after each cycle so it decouples the solution of the occupation of the virus in the cells from the previous iteration (Ψ^I^(*m*) from Ψ^R^(*m*)). If Ψ^R^(*m*) is zero in the equation for the infection rate, the solution of the virus in the cells becomes trivial. We have analyzed the model represented by the solutions in Eqs (6–10) for the full range of probability *p*, from *p* = 0 to 1. We find that the effect varying *p*, the probability that the virus remains in the cell if not cleared by the immune response, is only a slight rescaling of the temperature parameter, demonstrating universality in the solution described below. We also note that the addition of *p* breaks the symmetry between reproduction and infection but does not change the results. Since a value of *p* = 1 represents the most strongly coupled case of interaction between cells and virus, we present those results below.

#### Solution for the viral genetic states

Eq (6) (solutions to Eq 3) provide the viral occupation (or load) in the cells as a function of the distribution of virus in the environment. We next solve for the steady state distribution of virus in the environment. Imposing self-consistency on the reproduction and mutation processes, we derive the following equation (see S1 Appendix for details):

$$MD_{m}P_{m}=P_{m}[\sum_{m}D_{m}P_{m}]$$ (7)

Where ***M*** is a matrix of probabilities formed from Eq (5) and:

$$D_{m}=\frac{e_{m}^{2}(1-\Xi_{m})}{\left[1-(1-\Xi_{m})(1-e_{m})p\right]}$$ (8)

Solving Eq (7) gives the *steady-state viral probability distributions of the system*. Eq (7) can be recognized as an eigenvalue equation where every valid eigenstate *P*_*m*_ of matrix ***M*** *D*_*m*_ must have eigenvalue$\sum_{m}D_{m}P_{m}$. It can be proven that any eigenvector solution of ***M*** *D*_*m*_ *P*_*m*_ = *λ*_*m*_ *P*_*m*_ has an eigenvalue *λ*_*m*_ equal to $\sum_{m}D_{m}P_{m}$ as long as the eigenvectors *P*_*m*_ are normalizable as probability vectors (i.e.,$\sum_{m}P_{m}=1$) (note that *D*_*m*_ is expressed as a diagonal matrix).

Eq (7) defines a complex effective fitness landscape for the quasispecies population. We note that this fitness landscape is not purely multiplicative. Tripathi et al. (and references therein) modeled RNA virus evolution in a non-multiplicative fitness landscape where they allow for epistatic interactions [20]. However, the landscape considered here cannot simply be expressed as a sum of independent selection effects with cross terms.

#### Number of viruses

For each solution *P*_*m*_ of Eq (7), the number, *N*, of viruses in the environment is equal to the total probability that a cell has a virus that successfully reproduces, times the number of target host cells, *c*, times the fecundity, φ, defined above:

$$N=c\varphi \sum_{m}e_{m}\psi^{\Xi }(m)$$ (9)

*N* can then be found as the solution of a pair of coupled transcendental equations, one for *N* as a function of the infection rate λ, and the other for λ as a function of *N*. Substituting *Ψ* ^*Ξ*^ into Eq (9) from Eq (6), and using the definition of *D*_*m*_ from Eq (8), we obtain:

$$N=\lambda (N)\frac{c\varphi \sum_{m}D_{m}P_{m}}{E[1+\lambda (N)\sum_{m}K{'}_{m}]}$$ (10a)

with

$$K{'}_{m}=\frac{P_{m}e_{m}(1-\Xi_{m})(1-e_{m})p}{E[1-(1-\Xi_{m})(1-e_{m})p]}$$

$$\lambda (N)=\sum_{n=1}^{c}\frac{n}{c}{\left(1-E\right)}^{\left(c-n)(N-n\right)}\prod_{i=0}^{n-1}\frac{\left(1-{\left(1-E\right)}^{N-i})(1-{\left(1-E\right)}^{c-i}\right)}{\left(1-{\left(1-E\right)}^{i+1}\right)}\;\Theta (N-n)$$ (10b)

With *E*, defined in the context of Eq (6), *(1-E)* is the probability of a cell not being infected in a single viral pass given the distribution of virus in the environment *P*_*m*_. Further detail concerning the infection rate, λ(N), in Eq (10b) may be found in S1 Appendix. In the calculations in this paper, the number of target host cells, *c*, was taken to be 5 and the fecundity, φ, was 20, giving a maximum *N* of 100 viruses replicated from the cells into the environment.

These coupled nonlinear equations were solved using Newton-Raphson methods. It can be shown that the functional form above results in one and only one solution for *N*, for each choice of initial parameters and input viral distribution *P*_*m*_.

#### Numerical solutions

There are 51 roots of the eigenvalue Eq (7), corresponding to the vector size of *P*_*m*_, (which is indexed by the different *m*, *m* = 0 to 50). More generally, given a genetic target of length G, there would exist G+1 roots. We employed a number of tests to determine which of the eigenstates are physical. Each eigenvector element represents a probability. The eigenvectors should not have imaginary elements, and after normalization they should not have negative elements. The Perron-Frobenius theorem states that a real square matrix with positive entries has a unique largest real eigenvalue and that eigenvector has strictly positive components. Our matrix meets the criteria required by Perron-Frobenius so the all-positive eigenvector corresponding to the largest eigenvalue provides the equilibrium solution.

The number of viruses corresponding to an eigenstate (Eq 10) must also be greater than or equal to zero. With these conditions, only one nontrivial physical eigenstate was found for any set of initial conditions (temperature, immunity, etc.). The trivial zero state (no virus) is always a stable solution. We looked for dynamic solutions numerically and did not find any for the system defined in this paper. The dynamic solutions always converged to the analytically derived steady state result.

## Results and Discussion

### Evolution of the Virus

**Fig 5:**
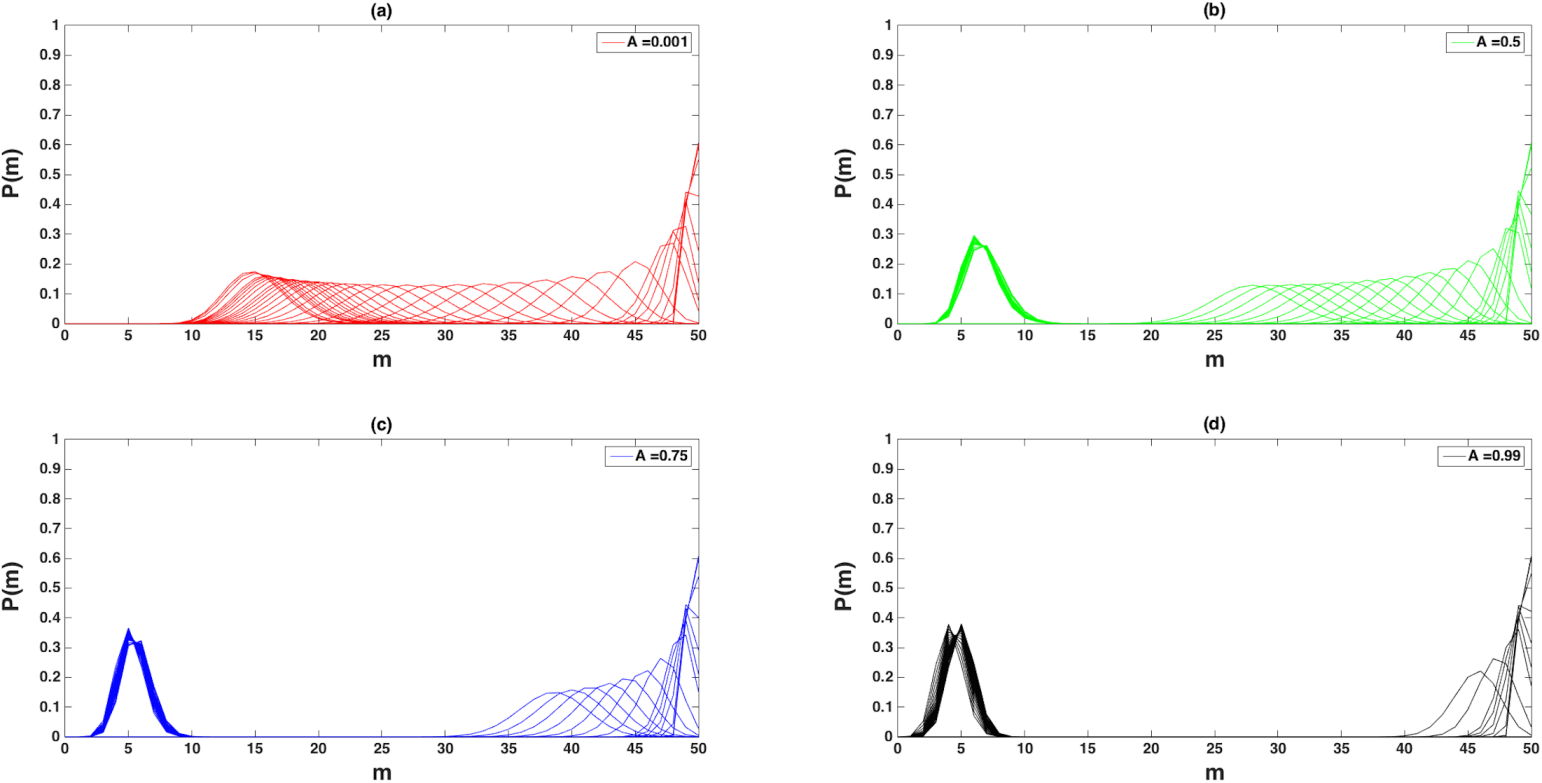
Eigenstates of the System. The figure shows the normalized eigenstates, P(m), vs. the number of matches, m, as a function of temperature and maximum immune response, A. Each distribution, P(m), represents the quasispecies distribution (i.e. probability of a given number of matches) at fixed A and temperature. The temperatures shown here are 0.01,0.03,0.05,0.1,0.3,0.5,1,2,3,4,5,10,…,100*,110,120,130,140,150,200,250,300 (*step by 5) for each immunity indicated. Low temperature is represented by the narrow distribution at high match (at far right).

The probability of a virus having a given number of matches, *m*, at a specific temperature and maximum immune response (Eq 4), A, is the normalized eigenstate, *P*_*m*_ (Fig 5). Each *P*_*m*_ can be thought of as the steady state quasispecies distribution, the peak of which represents the most “robust” virus type in the quasistates [14–16,20–22,30]. The width of each distribution reflects the accessible states and can be viewed as an indicator of evolvability or adaptive genetic diversity [4].

For all temperatures and immunities studied (with the exception of *T*, *A* = 0, see below), only one *stable* (non-trivial, non-zero) eigenstate was found. At very low temperature, the virus must closely match the target genome (*m* ~ 50). As temperature is raised, the mean number of matches of the quasistate decreases, eventually excluding the perfect match as an important component of the solution (i.e., the state de-pins from *m* = 50). Two distinct behaviors are observed as a function of immunity. At low immune amplitude (Fig 5A), as *T* increases, the mean of the distributions moves smoothly from high match to low match (*m*~14.5). At higher immune amplitude (Fig 5B–5D), the quasistates distribution jumps from higher to lower *m* with increasing *T*. This is most pronounced in Fig 5D where all eigenstates are found only near higher or lower *m* regions.

At low temperatures the virus must be well adapted to the host as reflected in the high codon match. Conversely, at very high *T*, the barrier is less important (i.e., entry into the cell is thermally “activated”) allowing the viruses to more easily avoid the immune system through greater genetic variation. We call distributions with mean near the perfect match “ordered states” of the virus, and distributions with low mean (*m*<10 in Fig 5D) “disordered states”. This suggests that the mean of the eigenstate distributions may serve as a measure of an order parameter for the system, that is:

$$M_{env}=\frac{1}{50}\sum_{m}mP\left(m\right)$$ (11)

An equivalent order parameter for virus *inside the cells* is defined in S1 Appendix.

**Fig 6:**
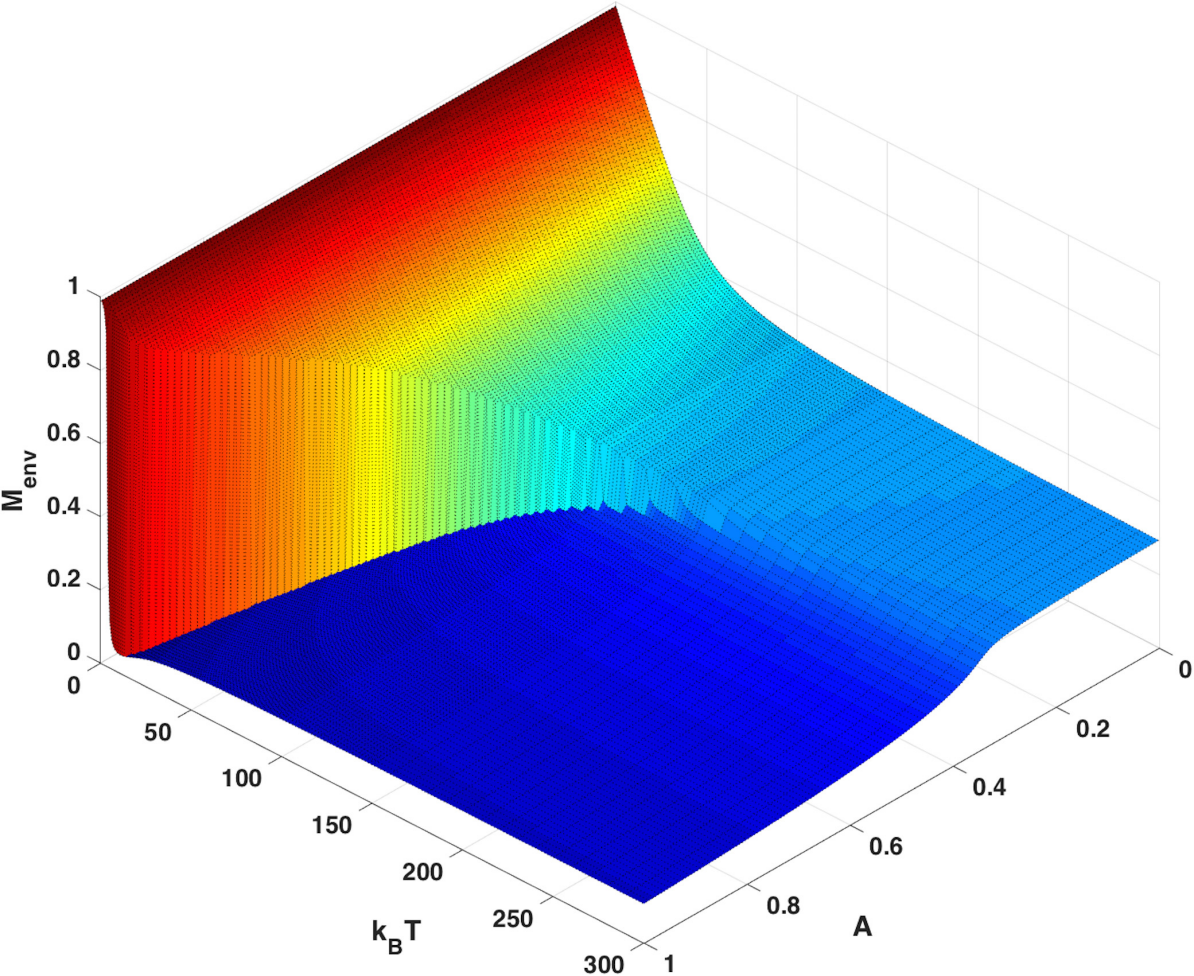
Order Parameter. The order parameter, *M*_*env*_, as determined by sampling virus in the environment to measure the average fraction of matching codons, as a function of temperature and maximum immune response, A. The order parameter is defined in Eq (11). Sampling virus inside the cells yields almost exactly the same result (see Figure C in S1 Appendix).

An order parameter, *M*, near 1.0 represents an ordered state and low *M* represents a disordered state. For low values of *A*, the order parameter decreases smoothly and continuously as temperature is raised (Fig 6). We will refer to this as the *regime of normal replication*. For high values of *A*, the order parameter jumps discontinuously from high to low as temperature is raised. This discontinuity suggests a first order phase transition in *T* at high immunity between the regime of normal replication and the disordered phase of the virus. The phase transition reported here reflects the competition between different natural “strategies” to resisting two different pressures. The first is immune response, and the second is the thermal barrier. Viruses responding to either of these pressures will have different characteristic energies. A first order phase transition occurs, by definition, when these energies cross. This phase transition is different from the phase transition that occurs in the Eigen and Schuster model, which reflects a loss in information associated with low fidelity of replication. It is also different from the very large literature on viral error catastrophe [14–22]. Error catastrophe is a phase transition that occurs in a dynamic model as mutation rate is increased past a critical rate. This is distinct from the phase transition observed above in the current steady state model. The phase transition observed here is a consequence of two competing energy terms. It is also distinct from the dynamic transition discussed by Nowak and May as a function of basic reproductive ratios for various mutant strains [16]. In our model even for increasing immunity in the disordered phase the viral population does not collapse.

**Fig 7:**
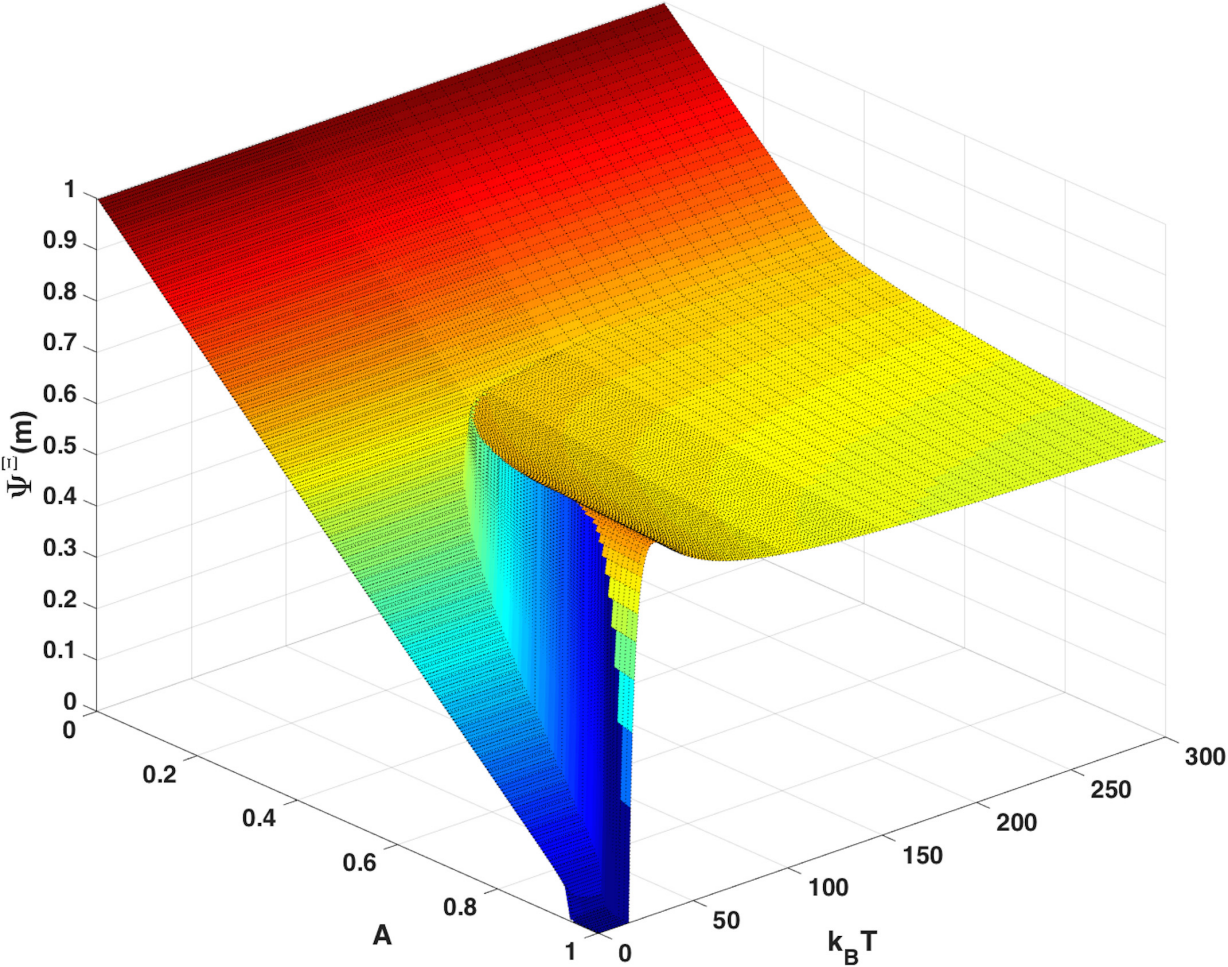
Occupancy of Cells. The occupancy of the cells, *Ψ* ^*Ξ*^ (m), derived from the steady-state solution (Eq 6), is shown as a function of temperature and maximum immune response, A. The same phase transition observed as in Fig 6 is evident here.

Fig 7 shows the *occupancy of the cells* after infection and immune response (before virus reproduction). One can view the occupancy as a measure of viral fitness. The occupancy fraction of the cells is between zero and 1. With zero immunity, A = 0, the cell occupancy is 1.0 for all *T*. As immunity is raised the occupancy decreases (approximately linearly in *A*) until reaching the phase boundary separating the regime of normal replication from the disordered phase. At high temperature and immune response the virus is in the disordered phase and cell occupancy plateaus at ~ 50%. These are viruses that never completely clear, but have low occupancy, low match, and evoke low immune response. At low temperature the virus never enters the disordered phase and cell occupancy decreases linearly with increasing *A*, eventually falling to zero. The region of phase space with zero virus (viruses that clear in steady state) appears small but for an individual with full immunity, it actually extends to T = 16 degrees which is 32% of the maximum mismatch energy (50) scaled by the effective Boltzmann constant. Likewise the region of zero virus is bounded by maximum immune response A> = 0.94. In the regime of large A and low temperature, one would expect a dynamic process with basic reproductive ratio below unity.

The discontinuity observed in Figs 6 and 7 is also evident in the order parameter measured for virus occupying the cells (see Figures C-D in S1 Appendix). These discontinuities suggest one or more phase transitions.

### Thermodynamics and Statistical Mechanics

To understand the possible phase transitions (Figs 6 and 7) we now study the thermodynamics of the system. To do so we must first define a temperature. So far, we have used parameter *T* as an “effective” temperature. At this point we go further and posit that *T* is in fact the natural temperature of the system. Systems at finite “natural” temperature do not stay in one equilibrium microstate. Rather, they sample all accessible states with a probability based on the Boltzmann distribution. We will estimate the effective Boltzmann constant and test the degree to which *T* acts as a real temperature below.

In order to determine the correct statistical thermodynamic ensemble of our system, we must identify the constant thermodynamic variables. In our model, the total number of cells, the size of the generic alphabet, and the length of the virus and the target genomes are all constant. To do thermodynamics, we need conservation of energy, and for this we need to define an energy. To be consistent with our definition of temperature, at *T* = 0 the system must enter a zero energy “ground state”. In our model, due to the Arrhenius form with temperature in the denominator of the exponential, at *T* = 0 the probabilities of infection and reproduction become delta functions at the maximum number of matches. Only viruses with a perfect match will successfully reproduce. We thus assign energy *E* = 50 − *m*. With this definition, at *T* = 0, only the *E* = 0 state of the virus will be present. Any multiple of *E* would also serve. A Boltzmann constant must relate the energy and temperature scales. That is, with this definition of energy, our denominator in Eq (3) is actually *k*_*B*_ *T*.

In general, the expectation value of the energy of a viral state with *N* total viruses in the environment at a given temperature and immunity is:

$$E=N\sum_{m=0}^{50}\left(50-m)P(m\right)$$ (12)

We calculate this and find the energy is zero all along the *T* = 0 axis for all immunities (as required), and increases monotonically with temperature. For reference, graphs of the expectation value of the energy and other thermodynamic variables may be found in S1 Appendix.

**Fig 8:**
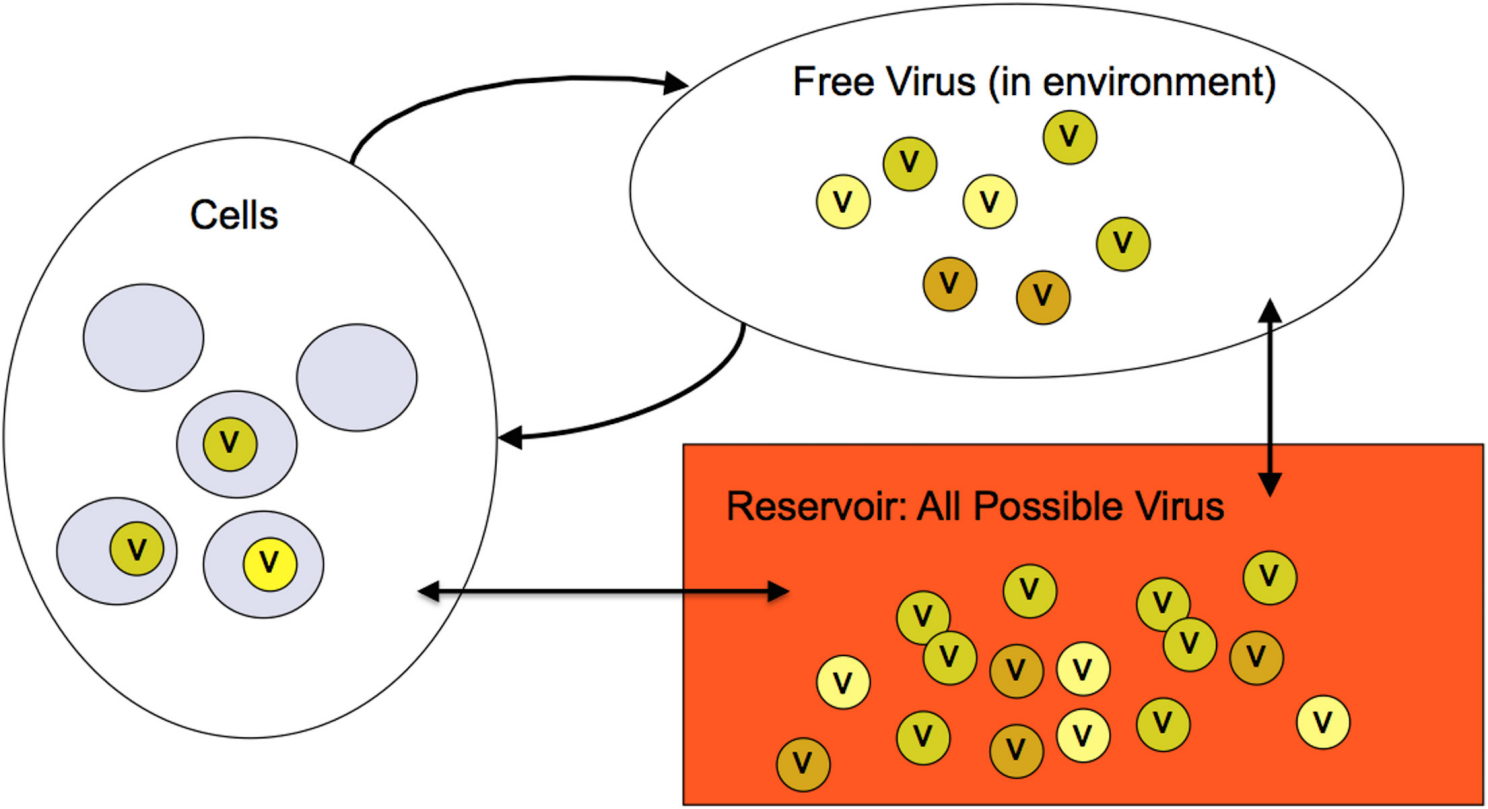
The Grand Canonical Ensemble for a System of Viruses. The three thermodynamic elements of the system are shown. The “Reservoir” of all possible virus is usually referred to as the “thermal bath”.[23] In this case the bath of possible viral sequences is very large (effectively infinite). Free virus in the environment, at steady state, is populated from the reservoir with a distribution based on temperature and immunity. In the infection phase, virus that successfully infect cells are drawn from the environment. Virus that fails to infect are returned to the reservoir. Immunity may remove virus (from the cells back to the reservoir), and reproduction draws new offspring from the reservoir and repopulates the environment (emptying the cells). The double arrows indicate population from and return to the reservoir. The curved arrows show the virus life cycle.

So far we have discussed our model in terms of viruses in the cells and in the environment. It is clear that as temperature and immunity are changed, both the energy and the number of virions change. Energy and number are both conserved *only* if we imagine that our cells and environment are *both* in contact with a third reservoir or *bath* that includes *all possible* viruses in thermal and “chemical” equilibrium with the rest of the system. Chemical equilibrium in our model requires conservative flow of virus between the reservoir and the system. Classically, for particle number to have an associated chemical potential, chemical potential of the system must be conserved during the internal dynamics of the system, and only able to change when the system exchanges particles with an external reservoir. This is the classic definition of a macro-canonical or *grand canonical ensemble* (Fig 8). This ensemble is the natural statistical ensemble for modeling any system of viruses. It ensures conservation of both number and energy. Any viruses not in cells or the environment (e.g., those eliminated by immune response) are in the reservoir, and any new viruses entering the system (e.g., mutated offspring) are drawn from the reservoir.

Consider an initial (fully occupied) state of the reservoir with no viruses in the cells or the environment. In this state the bath includes enough copies of all possible virus sequences to populate any possible system state. That is for each of the 26^100^ possible viral sequences there must be a number of copies equal to fecundity times the number of cells. Reproduction is then a process of drawing new viruses from the theoretical reservoir constrained by the rules of mutation. Since the total number of viruses is fixed, the total energy is fixed, thus assuring conservation of energy.

Given this ensemble it is possible to use the methodologies of thermodynamics and statistical mechanics to calculate any thermodynamic quantity of interest. For example, given the expectation value of the energy, the specific heat, *C(T)*, is defined by:

$$C(T)={\left(\frac{d\left\langle E\right\rangle }{dT}\right)}_{V}$$ (13)

where the derivative is taken at constant volume (here clearly maintained) and *<E>* is the average energy. Measurement of specific heat, or heat capacity, (shown in Figure J in S1 Appendix) is another indicator of the type of phase transition. We observe a sharp maximum in specific heat (i.e., a latent heat) in the vicinity of the apparent *first order* phase transition seen in Fig 6. We do *not* observe a power law singularity in that part of phase space where the system smoothly transitions between states, suggesting there is no second order phase transition.

**Fig 9:**
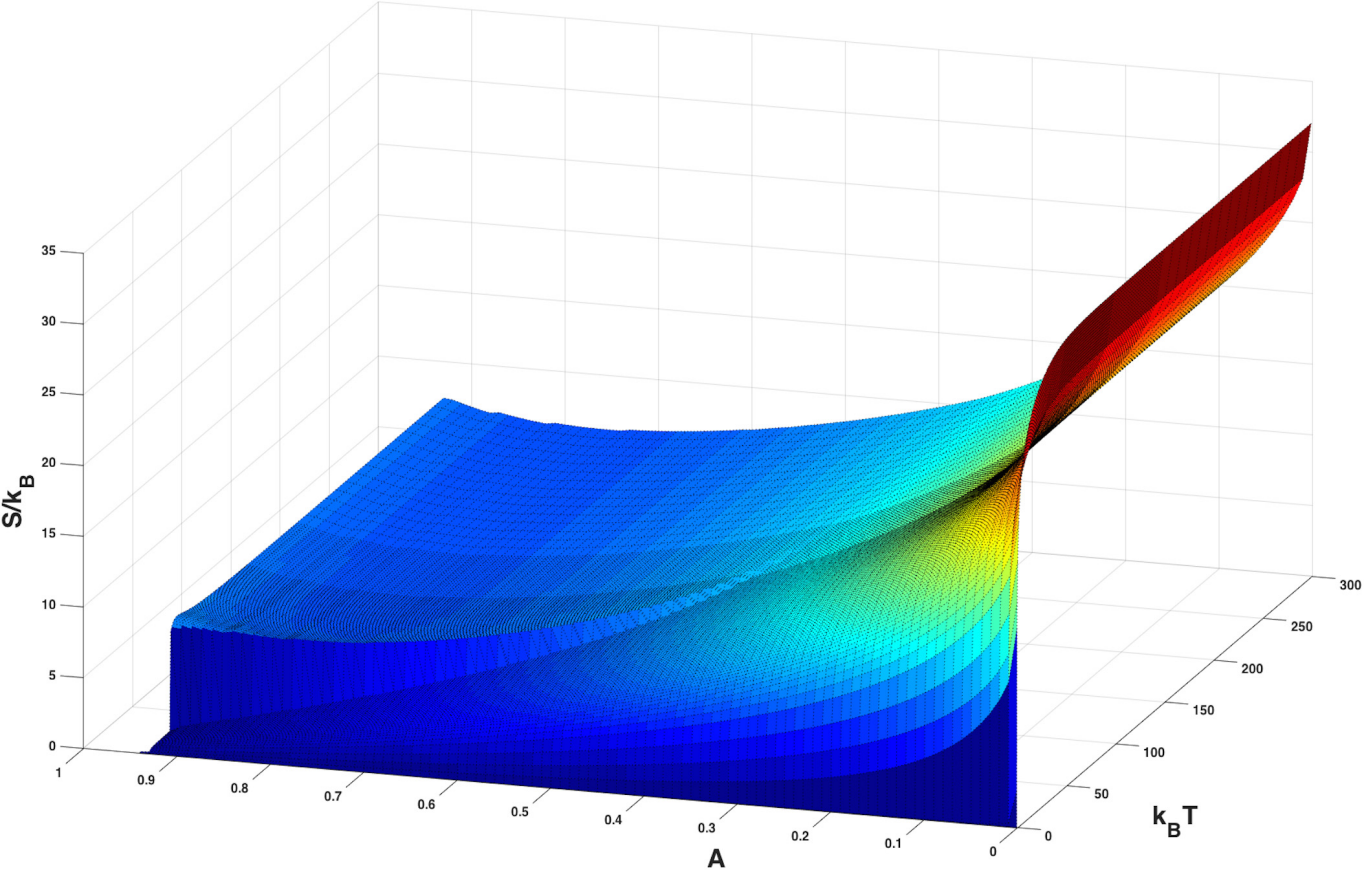
Entropy. The figure shows the entropy of virus, *S/k*_*B*_ *= ln Ω*, while in the cells as a function of temperature and maximum immune response, A, where *k*_*B*_ is the effective Boltzmann constant.

From the specific heat we also calculate entropy, a measure of the number of degrees of freedom of the system (Fig 9).

$$\Delta S=\int_{a}^{b}\frac{C(T)}{T}dT$$ (14)

Formally, the entropy is defined as *S* = *k*_*B*_ lnΩ, where Ω is the effective number of degrees of freedom in the system, and *k*_*B*_ is the effective Boltzmann constant. Here Ω is a measure of the genetic variability of the viruses. One can see from Fig 9, that the number of degrees of freedom is very large (as large e^35^ ~ 10^15^ even for our small genome). The number of degrees of freedom increases smoothly with decreasing peak immunity, *A*.

**Fig 10:**
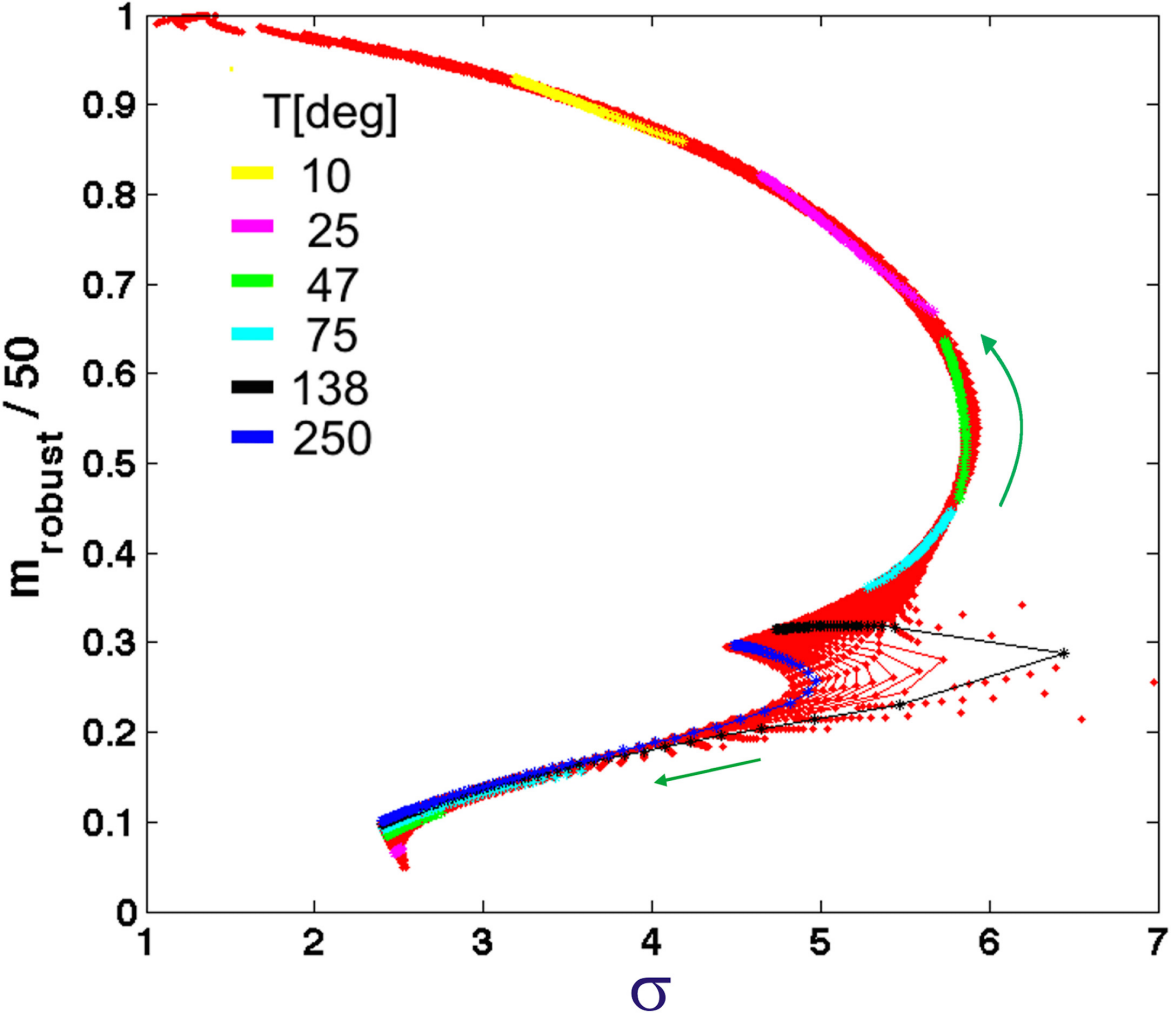
Robustness vs. Evolvability. The robustness (*m*_robust_/50) as a function of “evolvability”, σ, for each quasispecies, at all studied temperatures and immunities, *A* (red points). Here the robustness is defined by the order parameter, or average <m>, for each quasispecies distribution *P(m)*. For a symmetric distribution this corresponds to the most probable m. The curve is nearly universal, breaking apart only near the phase transition. The colored segments and the green arrows indicate the trajectories as function of increasing immunity (for temperatures represented).

In addition to calculating the entropy, we also obtained the *width*, σ, of each quasistate as a function of temperature and maximum immune response, *A*. As discussed above this width is a measure of evolvability or adaptive diversity. The order parameter, which corresponds to the most *abundant* number of matches in the quasistates is a measure of robustness, *m*_robust_ or the number of amino acids that can change without changing the match number or phenotype. In Fig 10 we plot *m*_robust_/50 as a function of evolvability for every temperature and immunity. Interestingly, we find that all of the data collapses onto a single universal curve. The evolvability is lowest for values of *T* and *A* that lead to quasispecies where *m*_robust_ closely matches the target as well as for quasispecies where the *m*_robust_ has almost no matches. The curve has a maximum for *m*_robust_/50 = 0.54. The largest evolvability corresponds to quasistates near the phase transition where the curve breaks apart.

In Fig 10 the segment in yellow represents the trajectory along the universal curve where immunity, *A*, is varied at fixed *T* (*T* = 10 degrees). Counterintuitively, as immune pressure is increased, evolvability decreases and m_robust_ increases. This behavior provides an explanation for the phase transition. In this model viruses must survive two types of pressure. Low temperature selects for phenotypes best adapted to infect the host. Immune response puts pressure on phenotypes that most closely match the target. The direction in which quasistate distributions shift in response to these combined pressures depends on the relative steepness of each energy term as a function of *T* and *A*. In the example at *T* = 10 degrees, with increasing immunity the most robust virus shifts to even better match the target thus lowering *temperature* driven barriers to reproduction. While this leads to a slightly higher immune pressure, the immune response function has nearly plateaued for matches above 15 codons, so there is diminished benefit from lowering the match to avoid immune response. As immune pressure is increased still further, there comes a tipping point where increasing visibility to the immune response becomes too much for the virus, and there is a jump in population characteristics–the phase transition–favoring a much lower match to the target. In general, for all of the states in Fig 10, all trajectories move away from the phase transition observed in Figs 5–8 (as indicated by the arrows in Fig 10). This behavior is further demonstrated by the shift in eigenstates as a function of immunity and temperature (see Fig 5 and Figure K in S1 Appendix).

The relationship between viral robustness and evolvability explored in Fig 10 deserves to be understood more in depth. Viruses, especially RNA viruses, use a number of strategies to preserve genetic information during replication. Neutral (synonymous) mutations, large population sizes, co-infections and molecular chaperones are just few of these mechanisms. On the other hand, adaptation through new mutations to harsh environments is paramount for the evolution of the virus. The apparent antithetic nature of these two necessary mechanisms implies the need for a tradeoff between them. Thus, a relationship like the one obtained in Fig 10 may to be necessary for the evolution of the virus. The observation that evolvability is at a minimum when robustness is very low or very high and at a maximum for intermediate robustness was first reported by Draghi et al. in a dynamic genotype-phenotype network model [12]. Stern et al. further confirmed both theoretically and experimentally the relationship between evolvability and robustness and observed this proposed universal behavior in polio virus [13].

### Thermodynamic Temperature

All of the analyses discussed above relate thermodynamic variables to strength of the immune system and an effective temperature. The question remains: how does our effective temperature relate to a real thermodynamic temperature? To determine this relationship, we calculate how the (genetic) states of the virus are distributed in energy.

$$\frac{1}{k_{B}T_{thermo}}\equiv \beta \equiv \frac{\partial \ln\Omega (E)}{\partial E}$$ (15)

where Ω *(E)* is the number of *accessible* states at energy *E*. The accessible states represent the entire cohort of N viruses. In the previous sections we calculated the equilibrium viral state and its properties as a function of effective temperature (*T*) and immune strength (*A*). At a given effective *T* and *A*, each state has a well-defined number of viruses, *N*, and a probability distribution, *P*_*m*_, representing the number of genetic matches (and mismatches) between the virus and target. While *N* and *P*_*m*_ are sufficient to calculate average properties (e.g., average energy), in order to calculate thermodynamic temperature one must enumerate the complete set of realizations of all systems with *N* viruses, and probability of match distributed as *P*_*m*._ In order to calculate Ω *(E)*, we need to do a careful counting of states as a function of energy.

We transform the probability distribution as a function of matches m, P_m_, into a probability of finding a virus at an energy *E*, *P(E)*, using the definition of Energy in Eq (12). Note that contributions to the probability of a virus at a given energy can be from several different quasistates. Details of how *P(E)* is calculated appear in S1 Appendix.

With the determination of *P(E)*, we can define the accessible states in energy as:

$$\ln\Omega (E)=\ln P(E)+\frac{\sum_{j=1}^{n_{E}}P_{j}(E)\ln D_{j}(E)-P_{j}(E)\ln P_{j}(E)}{P(E)}$$ (16)

with

$$\ln D_{j}(E)=\sum_{i=1}^{w_{j}}n_{ij}[\ln\Omega_{o}(m=m_{i})]$$ (17)

where Ω_o_(*m*) is the number of distinguishable configurations of the codons for a virus with m matches. This very large number depends on the number of matches, length of the virus and target genome length, the size of the alphabet, and the number of codons used (and not used) in the target. In addition, to be accessible, the states must be connected by permissible mutations. In practice this limits Ω_o_(*m*) from being the maximal value obtained by permutation alone. We have computed Ω_o_(*m*) numerically and find l*n* Ω_o_(*m*) ~ 47 for all *m*, given our definition of genomes, codon alphabet, and mutations.

In thermodynamics the formal relation between entropy and number of states is:

$${\text{S}=k}_{B}\;\ln\Omega$$ (18)

Note that in Eq (17) each contribution to lnΩ is of the form *p lnp*, which is the information theoretic entropy [31]. With these definitions we show below our calculated thermodynamic temperature as a function of the temperature parameter in our model, *T*_model_. From Eq (15) the effective *k*_*B*_ *T*_*thermo*_ is the inverse slope derived from a plot of lnΩ(*E*) vs. *E*.

**Fig 11:**
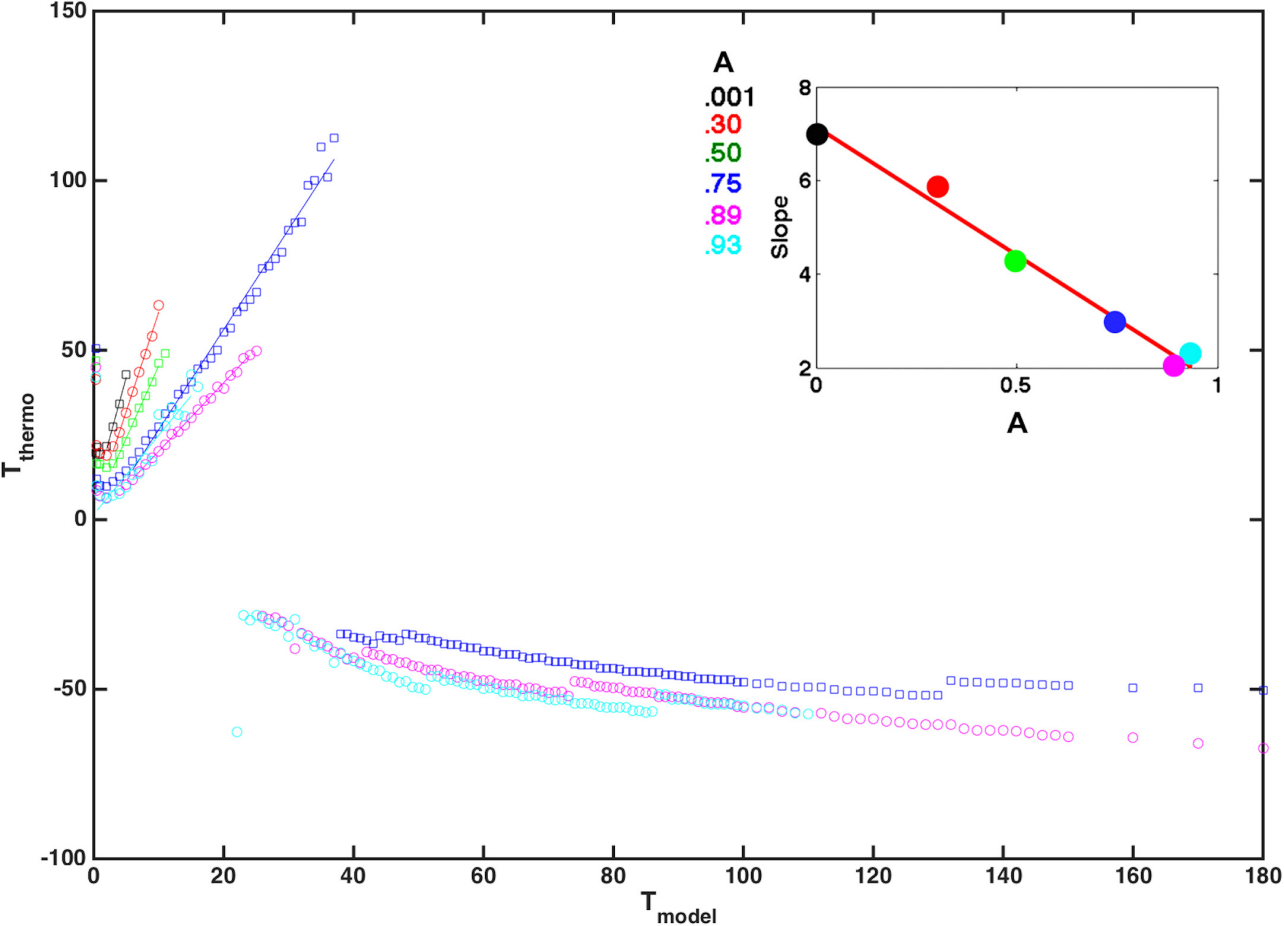
Testing for a Thermodynamic Temperature. The figure shows thermodynamic temperature *T*_*thermo*_ vs *T*_model_. For *T*_model_ below the phase transition, the relationship is linear with slope *k*_*B*_. From Eq (15) the *effective k*_*B*_ *T*_*thermo*_ is the inverse slope derived from a plot of lnΩ(*E*) vs. *E*. Above the phase transition a negative temperature is observed as expected. In the inset we plot the slope of *T*_*thermo*_ vs *T*_model_ for the values of maximum immune response, A, shown in the color legend, and observe that they all fall on a single line. This suggests that the effective *k*_*B*_ decreases linearly with increasing immune amplitude, *A*, and shows that immune strength rescales temperature.

For *T*_model_ less than the critical temperature (Figs 5 and 6), the system is in a regime of normal replication. In this phase, Fig 11 demonstrates that the thermodynamic temperature is defined, positive, and approximately linearly related to *T*_model_. The constant of proportionality is the *effective* Boltzmann constant. Observe that the temperature scale set by the entropy and the number of states is tied to the genetic properties of the virus+host *target pair*. A different type of virus with a protein receptor of different length or different degeneracy, or a different target (host) receptor would change the entropy and the energy and therefore the corresponding temperature scale. Lowering temperature can cause some virions to fail to infect any cell (in a particular host) which might otherwise been able to infect a cell. This is analogous to changing immunity, which can cause some virions to die, which may not have otherwise died. Changing temperature changes a virion’s fitness and shifts the steady state quasispecies distribution.

Viruses compete to infect a finite number of cells. Fitness of a quasispecies distribution is affected by temperature, immunity, and also the number of possible states at each match. There are many more virus genetic configurations with 15 matches than there are with 50 matches. So entropy plays a critical role in defining the thermodynamic temperature (and the steady quasispecies distributions). As illustrated in Fig 5 (A) with near zero immune response (for example) changing temperature causes the quasispecies distribution to shift.

In the regime of normal replication we find that the effective Boltzmann constant also depends on the immune strength, *A*. In the inset to Fig 11,we plot the slope of the thermodynamic temperature, *T*_*thermo*_ vs *T*_model_ for the immunities shown in the color legend, and observe that they all fall on a single line. This suggests that the effective *k*_*B*_ decreases linearly with increasing maximum immune response, *A*, and shows that immune strength rescales temperature in the regime of normal replication. This rescaling is approximately linear with *k*_*B*_ = -5.5*A* +7.2, where *A* is maximum immune response. We note with interest that for *A = 1* we have *k*_*B*_ *~2* implying the thermodynamic and model temperature scales are not far apart.

At the critical temperature, *T*_c_, there is a phase transition and the system switches from the regime of normal replication to a disordered phase for *T*_model_>*T*_c_. For reference, at high temperature and high immunity, the order parameter approaches zero and is nearly flat in the disordered phase. In fact, temperature is negative in the disordered phase. This negative temperature phase exists because at sufficiently high *T*_model_ there is less advantage to configurations with many matching codons, and at high immunity there is a survival penalty for eigenstates with high matches. Although it is possible to increase *T*_model_ to arbitrarily large values, the number of mismatches can never exceed the length of the target genome and the degeneracy of a state with maximal mismatch is constrained by the finite length codon alphabet. In classic textbook examples [23], Ω(*E*) is a rapidly increasing function of *E*. In this system, however, lnΩ(*E*) has a maximum near the phase transition and then decreases. This occurs because as temperature increases past *T*_c_ there are actually fewer accessible states in the disordered phase. This gives rise to a negative slope of lnΩ(*E*) vs *E* and, therefore, a negative temperature at high *T*_model_. Physically, negative temperature occurs any time a finite system has both an upper and lower bound to the *possible* energies. This is precisely the case in any system with finite length genomes and a finite codon alphabet. Theoretically this should also be true of real biological viruses but it remains to be seen if any examples exist.

Negative temperate defines the highest energy state(s) of a system. The current biologically inspired model provides an easy to understand example of why a state with negative temperature is hotter than a state at positive temperature. Temperature is defined not only by a kinetic energy but also by the total *entropy* of the system. In an infinite system, entropy increases as temperature is raised. In this finite biological system, as energy is increased past the critical point, entropy actually decreases because the number of possible states or configurations with no matching codons is always less than the number of possible states at lower energy with (e.g.,) one matching codon. A fully disordered *state* cannot use any of the codons found in the target so it has lower entropy. In the limit of very high energy (and negative temperature) the disordered *phase* represents a state with a cohort of viruses, some with no codons that match the target genome. Due to mutation the cohort must contain some offspring in the environment with some matching codons.

## Conclusions

In this paper we explored a simplified model of viruses and their life cycle. Within the model, the process of viral transmission is characterized by a series of energy barriers. A virus’s ability to cross these barriers is defined by its genetic similarity to an idealized target sequence for the host. The genetic properties of the viruses evolve, through natural selection, to a steady-state distribution of genetic states best adapted to an environment at each fixed temperature and immune response. The immune response represents the host’s ability to clear a virus based on both viral genetics and host immune memory. Viral evolution, in this case, is simply an operation on the genetic code of the multiple offspring of a parent virus.

The diversity of viral sequences in the extant population depends on the temperature of the system, *T*, and the strength of the immune response. At each temperature and immunity we find one stable quasi-state with a diverse distribution of viral sequences. The average of this distribution has a characteristic number of codons which match the target. This average match (*M*) defines an order parameter, and is found in our thermodynamic analysis to be related to the system energy. The width of the quasi-state distributions is a measure of the diversity (and evolvability) of the extant population. We find a nonlinear function relating evolvability to robustness that collapses all data at all temperatures and immune function to a single universal curve in agreement with previous theoretical and experimental literature [12,13].

We determined all equilibrium states of this model system, as well as the probability distribution describing the matches of those viruses as a function of temperature and immune response. The stable quasi-states and resulting virus phases as a function of immune response reflect the “strategies” a virus may take to efficiently infect a host cell while avoiding removal by the immune system. The order parameter based on the number of matches reveals two regimes. To understand these regimes we applied the machinery of thermodynamics and statistical mechanics. Enumerating the states of *all* possible viruses (those able to infect and reproduce in cells, off spring found in the environment, and a “reservoir” or “bath” of all remain states with their respective probabilities) we used the grand canonical ensemble to derive all of the thermodynamic variables for the system including thermodynamic temperature, immune suppression, entropy, specific heat, and total energy. The grand canonical ensemble is the natural statistical ensemble for modeling any system of viruses.

In response to temperature and immune pressure we observe a phase transition between a positive temperature regime of normal replication and a negative temperature “disordered” phase of the virus. In this model viruses must survive two types of pressure. Low temperature selects for phenotypes best adapted to infect the host. Conversely, immune pressure is strongest on phenotypes that most closely match the target. The direction in which quasistate distribution shifts in response to these combined pressures depends on the relative steepness of each energy term as a function of *T* and *A*. At some temperatures and immunities increasing immunity causes the virus quasistates to shift to even better match the target thus lowering *temperature* driven barriers to reproduction. As immune pressure is increased still further, there comes a tipping point where increasing visibility to the immune response becomes too much for the virus, and there is a jump in population characteristics–a phase transition–favoring a much lower match to the target.

The phase transition separates a regime of normal reproduction from a disordered regime with negative temperature. The negative temperature regime requires a scenario wherein a virus with few matching segments is still able to enter the cell. In real viruses there are many cases where we see large genetic diversity in individual genes, often those that are important to the immune response (e.g., surface proteins on hepatitis C virus and influenza). The action of these genes may be functionally more like a diffusion process, allowing greater diversity in the genes than would be expected in the regime of normal replication.

We have demonstrated that this simple model of viral replication has a real thermodynamic temperature linearly related to the effective model temperature where temperature is positive (thus defining the effective Boltzmann constant). Many important relevant modifications to the model and its parameters simply rescale the temperature. This suggests that if the model can be extended to capture the dynamics of true biological systems, complex aspects of such systems may similarly be understood using the formalisms of thermodynamics and statistical mechanics, thus greatly simplifying their analysis. Microbiological experiments systematically measuring the functional sensitivity of particular genes to changes in sequence may help to define the temperature scale of those genes and serve as an important step in adapting this model to real systems.

## Supporting Information

S1 Appendix The supplement contains additional derivations and detailed results. (PDF)
