# Supplementary material for: Statistical Mechanics and Thermodynamics of Viral Evolution
Source: PLoS One. 2015 Sep 30;10(9):e0137482. doi: 10.1371/journal.pone.0137482 (PMC4589373; doi:10.1371/journal.pone.0137482)
Supplement: S1 Appendix — (PDF) [file pone.0137482.s001.pdf]

Statistical Mechanics and Thermodynamics of Viral Evolution
Supplemental Material: S1 Appendix

Viral Infection as Energy Barriers

The process of infection depends on the ability of a virus to successfully bind to and enter a target host cell [2,3]. As discussed in the manuscript, we treat the cell membrane (and structures within it) as a "barrier" to entry of the cell. Using an activated Arrhenius form, we model the temperature dependence of reaction rates to compute the probability of crossing this barrier [5]. Viruses well adapted to a particular host are most able to overcome the barrier, enter the cell, and subsequently use the cell's genetic factory to reproduce. We *abstract* the fitness of a virus with respect to a target host barrier in terms of how well a genetic code within the virus "matches" the part of the host cell's genetic code that encodes for the binding sites that allow or block entry. As shown in Fig A, we represent this code as an alphabetic string and interpret genetic similarity in terms of the number of alphabetic character in the virus "genome" that match the target characters in the host genome. We define *m*, the "number of matches," as the largest number of locations at which the virus genome can match the target genetic sequence for any alignment of virus with target (which completely overlays the target). "Temperature" plays the role of a discriminator. At low temperature only viruses with a near exact match with the target can pass, while at high temperatures all viruses have a good chance of passing. Since we have abstracted the real chemistry, the "effective temperature" at this point can be viewed as a tuning parameter that adjusts the binding process and its effectiveness in discriminating between foreign viruses. We later demonstrate that this tuning parameter is the thermodynamic temperature for the system, and we derive the effective Boltzmann constant relating energy to temperature.

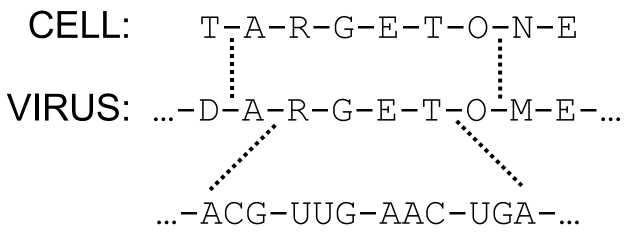

Fig A. The fitness of a virus relative to a host depends on the genetic code of both the virus and the host. In our simple model we represent this symbolically by alphabetic characters that in turn represent genetic codons.

Self Consistency

As an example of the self-consistent nature of the system, consider the possible states of the system post-infection (manuscript Fig 2). Whether there is a virus in the cell or not

can give rise to considerably different decision trees. For self-consistency to hold, the state reached post-infection from this cycle must be the same as that started from. The decisions portrayed in Fig B are simplified, in that they do not show the distribution of viruses (in genetic space). Moreover, and this is an important point which will hold for all of the analytic expressions to follow, all cell occupations are probabilistic and can take any value from zero to 1; likewise the free viruses are characterized by a probability distribution of matches.

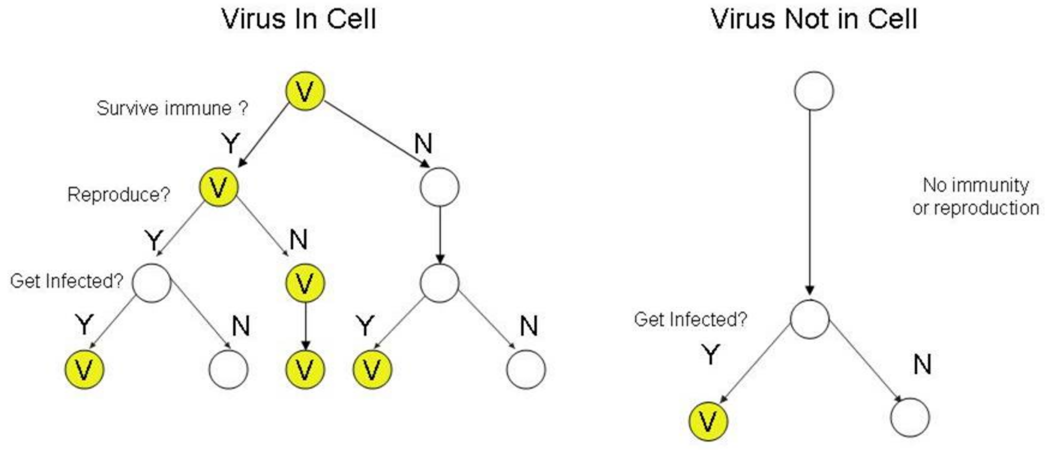

Fig B. Sample decision trees, based on averaged distributions of viruses, for virus survival at each stage of the life cycle, starting at the post-infection stage. After just one iteration to the next infection stage, there can be quite a complex distribution of possible states. The goal is to reach a steady-state solution.

Model Infection Process

With the criteria defined in the manuscript, we have analytically derived the overall infection rate. Below we give detail on several implications of this equation. As in the manuscript, Eq (10b), we define the infection rate, λ, as the probability a cell is infected as a function of the number, N, of free viruses in the environment.

$$\lambda(N) = \sum_{n=1}^{c} \frac{n}{c} B^{(c-n)(N-n)} \prod_{i=0}^{n-1} \frac{(1-B^{N-i})(1-B^{c-i})}{(1-B^{i+1})} \cdot \Theta(N-n) \qquad \text{(S1)}$$

where c is the number of target host cells, and B is the probability of a cell *not* being infected in a single viral pass given the distribution of virus in the environment P_m.

$$B = 1 - \sum_{m} e_m P_m \qquad \text{(S2)}$$

Eq (S1) is only valid for integer numbers of free viruses N. In practice, however, just as all viruses in the environment are represented by a vector of probabilities P_m for their distribution among all the *m*'s, the total number of viruses N is likewise a real number in this model. A non-integer value of N, with integer part n_0, can be viewed as a probability $N - n_0$ that the number of viruses is $n_0 + 1$, and probability $n_0 + 1 - N$ that the number of viruses is n_0. It can easily be seen that N represents the most probable value in this case (i.e., the "expectation value").

To calculate the overall infection rate $\lambda(N)$ for non-integer N, therefore, we calculate

$$\lambda(N) = (N - n_0)\lambda(n_0 + 1) + (n_0 + 1 - N)\lambda(n_0) \qquad \text{(S3)}$$

where λ on the right hand side is evaluated using Eq (S1) at the integer values n_0 and $n_0 + 1$ as shown in Eq (S3).

Solution for the Viral Genetic States

We derive the processes that occur when a virus reproduces. In the current model, when a virus reproduces, the cell dies, and a new empty (uninfected) cell takes its place. The distribution of free viruses changes significantly upon reproduction. In this model, the virus reproduces with a fecundity, $\boldsymbol{\varphi}$, where we set

$$\boldsymbol{\varphi} = 20. \qquad \text{(S4)}$$

Each offspring is mutated at a randomly chosen single codon. This collection of mutated viruses is added to the pool of "free virus" available for infection at the next iteration. We call this pool "the virus in the environment." As the system evolves toward steady state, these mutations enable the virus to adapt to the competing factors such as the adaptive immune response and the barrier to infection. The number of virus in the environment fluctuates iteration to iteration, until stability is reached. The analytic solution, Eq (6), yields only the equilibrium or steady-state solution. We also solved the equations numerically and iteratively to track the dynamic approach to equilibrium and test for dynamic instability.

After reproduction the probability of a virus remaining in the cells is $(1 - e_m)\psi^{\Xi}(m)$, and the probability to successfully reproduce is $e_m\psi^{\Xi}(m)$. We transform the probability functions above into a virus probability via

$$P^{premutation}(m) = \frac{e_m\psi^{\Xi}(m)}{\sum_m e_m\psi^{\Xi}(m)} \qquad \text{(S5)}$$

Each offspring is subject to the possibility of mutation (manuscript Equations 5).

$$P^{postmutation}(m) = \boldsymbol{M} P^{premutation}(m) \qquad \text{(S6)}$$

Here, $\boldsymbol{M}$ is a matrix of probabilities formed from manuscript Equations 5. Because the mutation can only change the number of matches by at most +/- 1, the resulting matrix is tri-diagonal.

In substituting $\psi^{\Xi}(m)$ from manuscript Equations 6 into Eq (S5), an important point arises. The P_m which appears in Equations 6 is in fact the post-mutation viral distribution from the previous iteration. Our stability criterion is that the post-mutation viral distribution must be constant, iteration to iteration. Setting the P_m from Eq (6) equal to $P^{postmutation}(m)$ in Eq (S6) and calling them both P_m , we obtain, finally, the self-consistent equation for P_m , the post-mutation stable viral distribution:

$$\boldsymbol{M} D_m P_m = P_m[\sum_m D_m P_m] \qquad \text{(S7)}$$

where

$$D_m = \frac{e_m^2(1-\Xi_m)}{[1-(1-\Xi_m)(1-e_m)]} \qquad \text{(S8)}$$

Eq (S7) can be recognized as an eigenvalue equation where every valid eigenstate P_m of matrix $\boldsymbol{MD}_m$ must have eigenvalue $\sum_m D_m P_m$. As it turns out, it can be proven that any eigenvector solution of $\boldsymbol{M} D_m P_m = \lambda_m P_m$ has an eigenvalue λ_m equal to $\sum_m D_m P_m$ as long as the eigenvectors P_m are normalizable as probability vectors (i.e., $\sum_m P_m = 1$). In evaluating the matrix product $\boldsymbol{MD}_m$ we note that D_m is expressed as a diagonal matrix.

Solving Eq (S7) gives the steady-state viral probability distributions of the system. The tri-diagonal matrix $\boldsymbol{MD}_m$ is *asymmetric*. In order to increase the numerical accuracy of the eigenvalue calculations, we used a similarity transformation on $\boldsymbol{MD}_m$ to form a symmetric tri-diagonal matrix $\boldsymbol{S}$ with the same eigenvalues.

The details of a general similarity transformation from nonsymmetric triadiagonal to symmetric tridiagonal matrix is shown as follows.

For a non-symmetric tri-diagonal matrix M with nonzero same-sign entries M_{ij}, the

transformation to a symmetric matrix $S = B^{-1}MB$ with the same eigenvalues as M is obtained by similarity transformation with a diagonal matrix B. To determine B, there is an overall scale factor that is left undefined. For convenience we set $B_0 = 1$. With this definition, the remaining elements of B are:

$$\begin{aligned} B_1 &= \sqrt{M_{10} / M_{01}} \\ B_2 &= B_1 \sqrt{M_{21} / M_{12}} \end{aligned} \tag{S9}$$

and in general

$$B_n = B_{n-1} \sqrt{M_{n,n-1} / M_{n-1,n}} \tag{S10}$$

We note that at a minimum for this transformation to be defined, off-diagonal pairs $M_{n,n-1}$ and $M_{n-1,n}$ must be of the same sign and nonzero. In closed form this gives:

$$\begin{aligned} B_0 &= 1 \\ B_n(n > 0) &= \prod_{i=0}^{n-1} \sqrt{\frac{M_{i+1,i}}{M_{i,i+1}}} \end{aligned} \tag{S11}$$

The resulting matrix S has

$$\begin{aligned} S_{ij} &= S_{ji} = \sqrt{M_{ij} M_{ji}} \\ S_{ii} &= M_{ii} \end{aligned} \tag{S12}$$

It can be easily shown that S defined in this manner has the same eigenvalues as M. The eigenvectors V of S are related to the eigenvectors P of the original matrix M by

$$\begin{aligned} P &= BV \\ P_n(m) &= B_m V_n(m) \end{aligned} \tag{S13}$$

It can be noted therefore that if the elements of V_n are real and positive definite, that the elements of P_n will be as well.

Besides being faster to evaluate, the eigenvectors of $\boldsymbol{S}$ had much cleaner distinctions between physical and nonphysical eigenvalues (see manuscript). Since the transformation from the eigenvectors of $\boldsymbol{S}$ to those of $\boldsymbol{MD}_m$ is real and positive definite, the physical eigenvectors of $\boldsymbol{S}$ correspond directly to the physical eigenvectors of $\boldsymbol{MD}_m$.

Order Parameter and Occupancy for General Values of p

As discussed in the text, Equations 6-10 can be solved for values of the probability p, the probability that the virus remains in the cell if not cleared by the immune response, with 0<=p<=1. We find that varying p leads only to a slight rescaling of the temperature parameter, T_{model}, demonstrating universality in the solution. Supplemental Figs C-D compares the order parameter at p=1 with p=0, the limit where all virus exit the cell whether they successfully reproduce or not. The phase transition is observed for all p with only a slight rescaling of temperature.

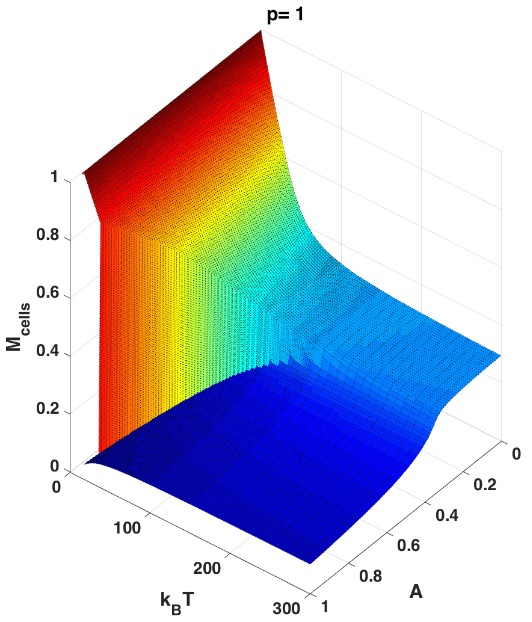

Fig C. Order parameter, M, measured in the cells vs. temperature, T, and maximum immune amplitude, A, at p=1 as in manuscript Fig 6.

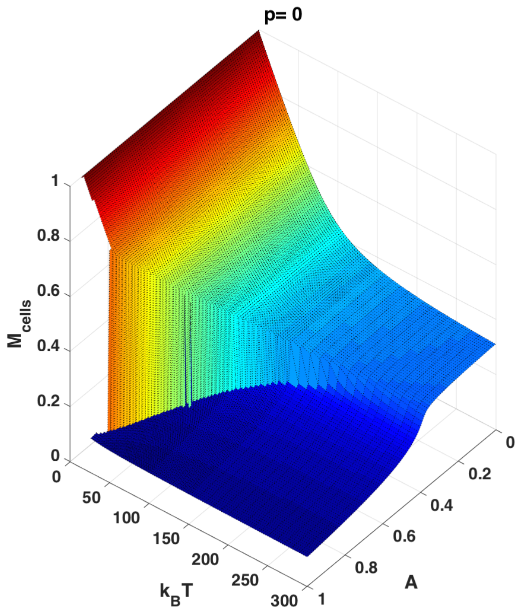

Fig D. Order parameter, M, measured in the cells vs. temperature, T, and maximum immune amplitude, A, in the limit p=0. Varying p leads only to a slight rescaling of temperature.

In Figs E-F we similarly compare the occupancy of the cells for p=0 and p=1.

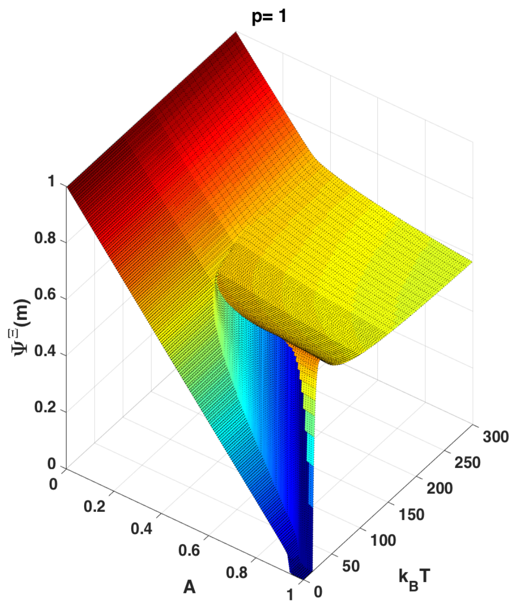

Fig E. Occupancy of the cells, Ψ^{Ξ} *(m)*, vs. temperature, T, and maximum immune response, A, at p=1 as in manuscript Fig 7.

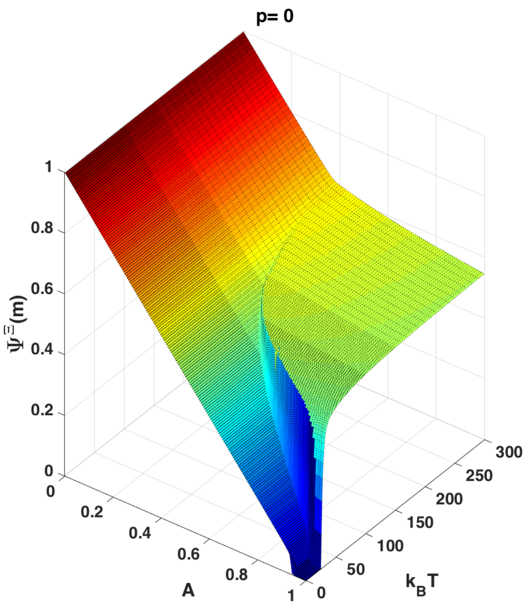

Fig F. Occupancy of the cells, Ψ^{Ξ} *(m)*, vs. temperature, T, and maximum immune response, A, in the limit p=0. Varying p leads only to a slight rescaling of temperature.

Fig G is a heat map of the data in manuscript Fig 6. The top down view makes it somewhat easier to see the separation between the normal binding regime and disordered phase a function of temperature and maximum immune response, A.

Also shown in Fig G is a parametric fit of the boundary between the regime of normal binding and the disordered phase in T,I. The function is a Gaussian (in T) centered at T=0 with a constant offset (in I)

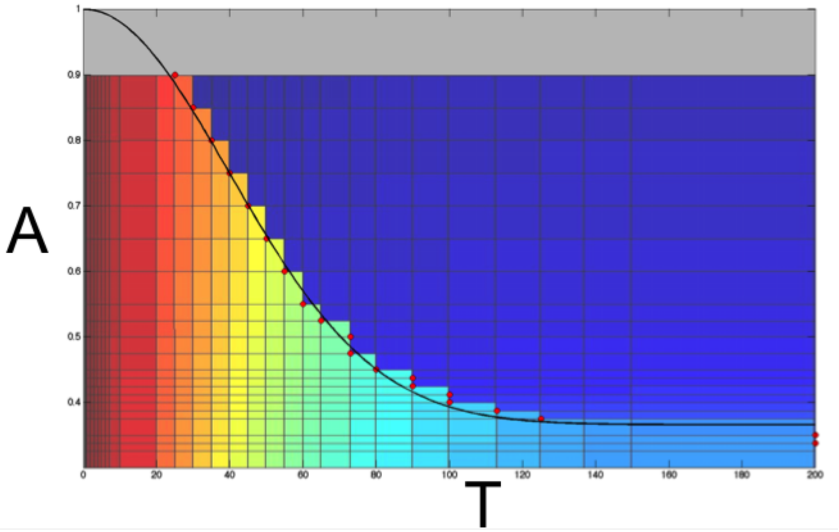

Fig G. Top down view of order parameter (manuscript Fig 6) showing the separation between the regime of normal binding and the disordered phase as a function of temperature, T, and maximum immune response, A.

$$I^*_{crit} = (1.0 - C) + Ce^{-(T^*_{crit})^2/2\sigma^2} \tag{S14}$$

with fitting parameters

$\sigma = 39.85$

$C = 0.634$

This boundary equation separates the regime of normal binding from the disordered phase, although the nature of the transition appears to change as Temperature is raised (and maximum immune response is lowered).

Virus in the Environment

Fig H represents the steady-state number of virus in the environment post reproduction for viruses under varying selective pressure based on the analytic solution to our eigenvalue Equations S7.

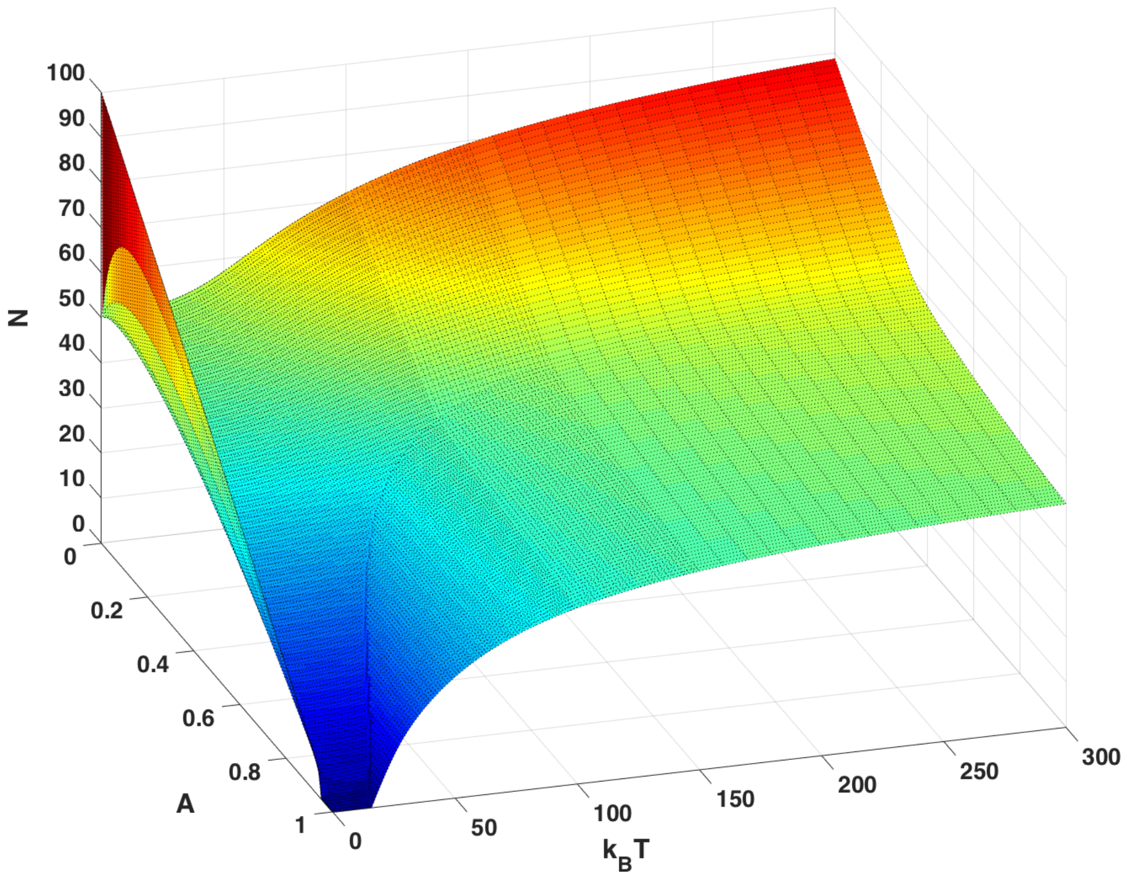

Fig H. Number of virus, N, (viral load) in the environment post reproduction as a function of temperature, T, and maximum immune response, A, from the steady-state solution.

The phase boundary observed in manuscript Fig 6 is also evident as an inflection point in Fig H. Also evident in Fig H is an upturn in the number of virus in the environment at T=0 near A=0. This is a singular point in our model which has different solutions depending on whether the system approaches T=0 or A=0 first. If A=0 and non-zero but small T, the steady-state solution in the cells is only the perfect virus. The probability becomes very narrow and peaked at m=50. The perfect virus has a reproductive advantage and no immune pressure. Approaching T=0 from this limit leads to the greatest possible number of virus. On the other hand, if T=0 and non-zero but small I, the steady-state solution is a 50/50 mix of m=49 and m=50. At every cycle the m=50 virus are cleared by the non-zero immune response but the m=49 virus reproduce, with mutation, filling the environment with a mix of m=49 and m=50. The number distribution is such that both successfully infect the cells on the next cycle. The mixed state remains stable approaching A=0 leading to the observed jump along T=0 axis.

Thermodynamic Variables

Temperature Scale

In this model the *effective* k_B has units of [matches/degree]. The temperature scale here begins at zero for the zero energy ground state, analogous to the Kelvin scale, but the scale itself is arbitrary (as is the energy of a "match"). It varies with effects like immune strength as discussed in the paper. In a physical system the binding energy would be specified in standard units (e.g. SI) and k_B and Temperature defined accordingly.

Average Energy

As discussed in the text, the expectation value of the energy of a viral state with N total viruses in the environment at a given temperature and maximum immune response is:

$$E = N\sum_{m=0}^{50}(50-m)P(m) \qquad \text{(S15)}$$

We plot this quantity in Fig I, and find all along the T=0 axis, the energy for all immunities is zero, as required.

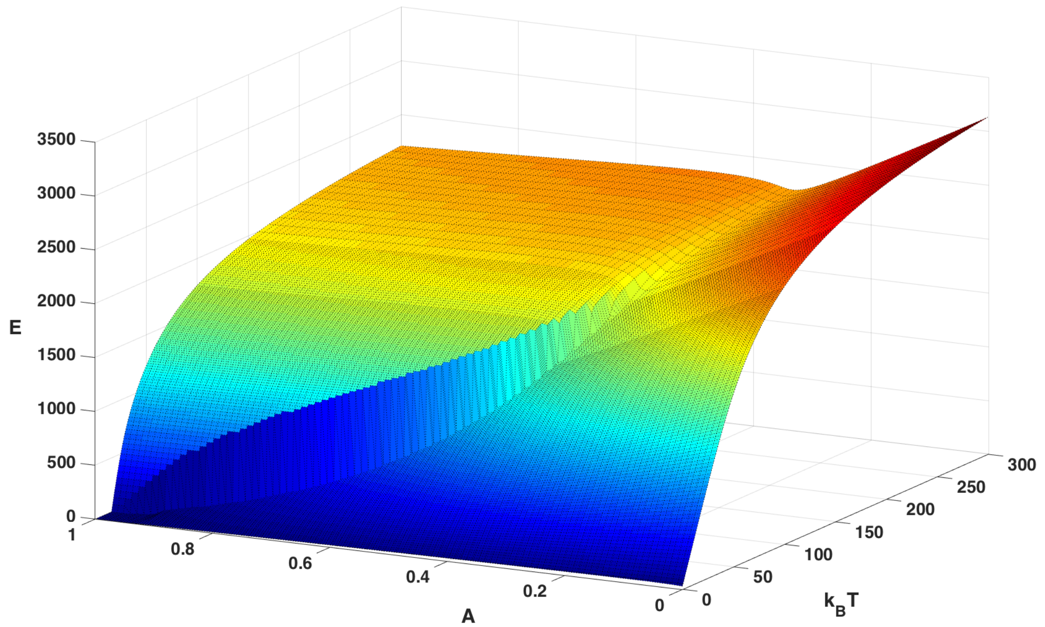

Fig I. Average Energy, E, of the Viruses in the Environment

Specific Heat

The main feature of the specific heat, defined in the manuscript by Eq (13), is the sharp peak along the apparent first order phase transition (Fig J).

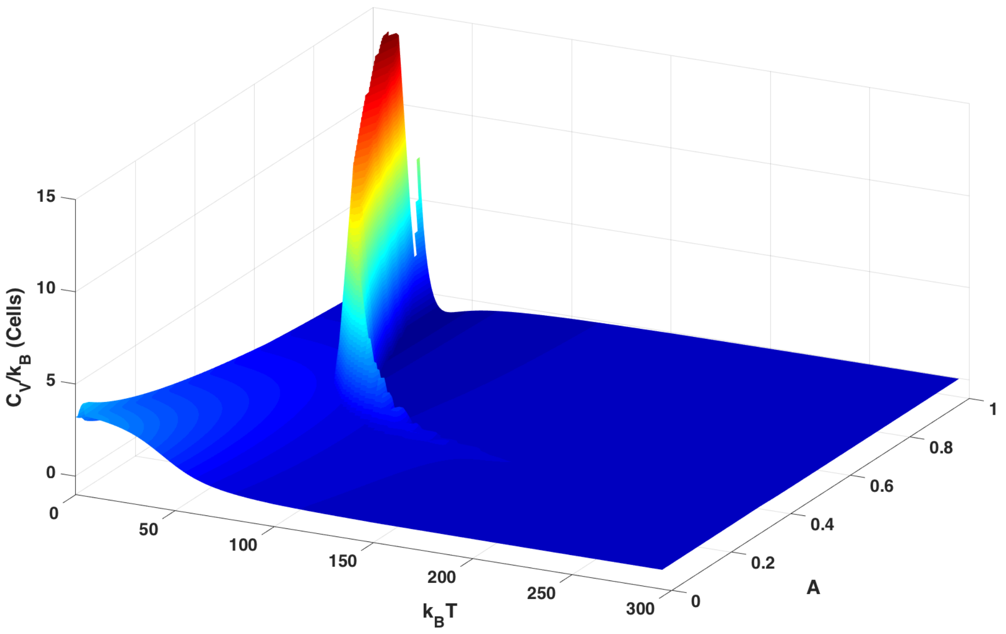

Fig J. Heat Capacity of the Cells, C_V/k_BT

Thermodynamic Temperature

To determine how effective temperature is related to real thermodynamic temperature, we calculate how the (genetic) states of the virus are distributed in energy.

$$\frac{1}{k_B T} \equiv \beta \equiv \frac{\partial \ln \Omega(E)}{\partial E} \qquad \text{(S16)}$$

where Ω (E) is the number of *accessible* states at energy E. The accessible states represent the entire cohort of N viruses. In the previous sections we calculated the equilibrium viral state and its properties as a function of effective temperature (T_{model}) and maximum immune response (A). At a given effective T_{model} and A, each state has a well-defined number of viruses, N, and a probability distribution, P_m, representing the number of genetic matches (and mismatches) between the virus and target. While N and P_m are sufficient to calculate average properties (e.g., average energy), in order to calculate thermodynamic temperature one must enumerate the complete set of realizations of all

systems with N viruses, and probability of match distributed as P_m. In order to calculate $\Omega(E)$, we need to do a careful counting of states as a function of energy. The first step is to translate the probability distribution P_m shown in manuscript Fig 3 as a function of matches into the corresponding distribution as a function of energy, $P(E)$. Where we define

$$P_j(E) = N_j! \prod_{i=1}^{w_j} \frac{P_{m=m_i}^{n_{ij}}}{n_{ij}!} \qquad \text{(S17a)}$$

and

$$P(E) = \sum_{j=1}^{n_E} P_j(E) \qquad \text{(S17b)}$$

$$\sum_{i=1}^{w_j} n_{ij} = N_j \qquad \text{(S17c)}$$

In the equations above, N_j, is the number of viruses in eigenstate j and, n_E, is the number of states at energy E. We define w_j as the distribution width of eigenstate j; n_{ij} is the occupation of the ith "match" bin (measured in number of viruses) for the jth eigenstate. With these definitions, we can define the accessible states in energy as in manuscript Eq (16).

As discussed in the paper, in order to calculate thermodynamic temperature one must enumerate all realizations of all systems with N viruses, and a probability of match distributed as P_m. This would include, for example, a state with N viruses all of which have zero matches but with very small probability. We note that it also includes states with mixed numbers of matches (every combinatorial possibility). For example, for N = 13, one possible state might have a single virus with zero matches, 4 viruses with 13 matches, and a final 8 viruses with 26 matches. The probability of this state is:

$$(P_{m=0})^1 \, (P_{m=13})^4 \, (P_{m=26})^8 \qquad \text{(s17)}$$

For the example above $n_{ij} = \{1,4,8\}$.

Even this state can be represented in several different ways depend on which of the 13 viruses have m=13 vs. m=26, etc. It is clear that even for moderate N, the number of possible combinations is large. In principle enumerating all the states requires 51 nested computational loops.

Fortunately, we observe (see manuscript Fig 5a-d) that all distributions, P_m, have a well-defined width. Given the finite distribution width, w, we can reduce the number of possible states by only considering a number of matches where the value of P_m is greater than a threshold, which we take to be 10^{-4}. With this approximation, no distribution is observed to have a width greater than 26 matches. Thus, the required computation can be done with a maximum of 26 nested logical loops (called by recursion).

Robustness vs. Evolvability

As discussed in the paper, we also plot the order parameter $m_{robust}/50$, a measure of the evolvability or adaptive diversity of each quasistate, as a function of evolvability measured from the width of the normalized eigenstates P_m. We find a universal curve (manuscript Fig 10) where trajectories in temperature and maximum immune response, A, lead away from the phase transition observed in manuscript Figs 5-7. In Fig K we show the normalized eigenstates as a function of immunity at several values of temperature shown inset. This figure is complementary to manuscript Fig 5. The figure indicates increasing immunity, A, by the colors progression black (low), yellow, red, green, blue (high). The phase transition is evident in Fig I as the "gap" between the ordered and disordered regimes at certain temperatures. From the color progression the figure demonstrates that the eigenstates or quasispecies distributions always move away from the gap as immune pressure is increased. At any T and A, there are two different pressures on the virus. Which direction the virus evolves in response to these combined pressures depends on the relative gradient of each environmental factor.

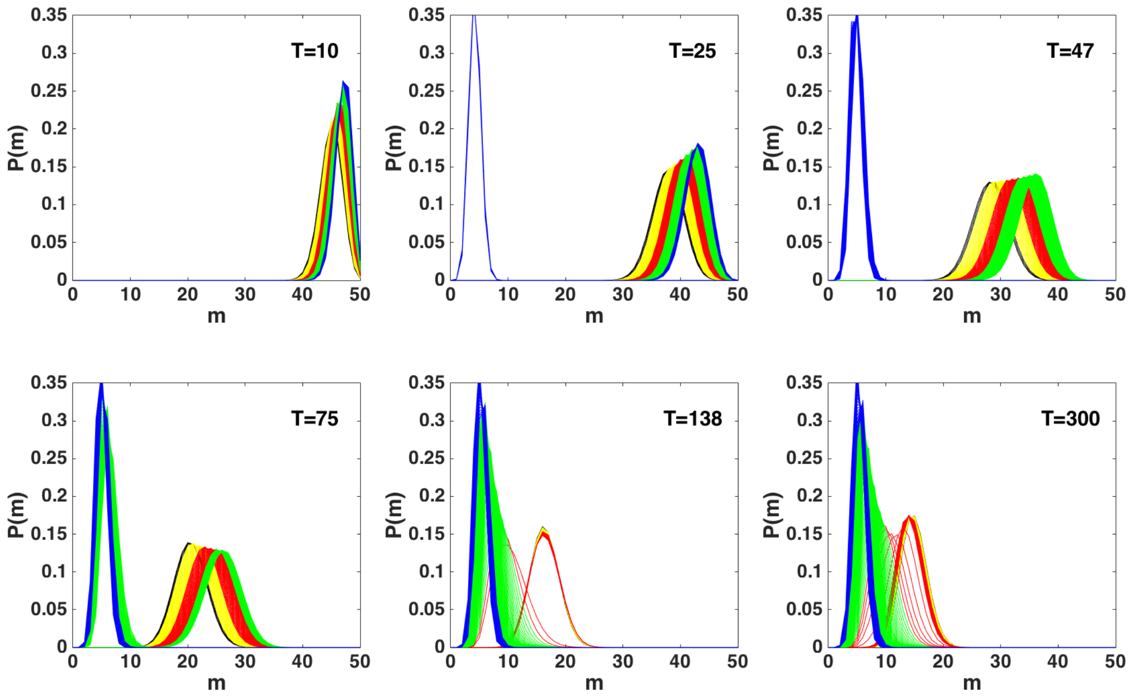

Fig K. The normalized eigenstates, P(m) as a function of maximum immune response, A, at several values of temperature shown in inset. Increasing immunity, A, are indicated by the color progression black (low), yellow, red, green, blue (high). From the progression with increasing A, the figure shows that the quasispecies distributions always move away from the gap as immune pressure is increased.
